# Supplementary material for: Cell States and Interactions of CD8 T Cells and Disease-Enriched Microglia in Human Brains with Alzheimer’s Disease
Source: Biomedicines. 2024 Jan 25;12(2):308. doi: 10.3390/biomedicines12020308 (PMC10886701; doi:10.3390/biomedicines12020308)
Supplement: Supplementary file 1 [file biomedicines-12-00308-s001.zip › Supplementary Figure legends.pdf]

Supplementary Figure legends:

Supplementary Figure 1. Composition of concurrent Lewy body pathology in different age groups.

Stacked bar plots of Lewy body pathology in 90 years of age or older cases and younger group. Y-axis represents proportions among total number of the cases in the group.

Supplementary Figure 2. UMAP plot for sample metadata of Microglia-perivascular macrophage (PVM). Seurat object as downloaded from SEA-AD: Seattle Alzheimer's Disease Brain Cell Atlas (40,000 cells from 84 samples) labeled for clusters, age at death, donor ID, specimen ID, PMI, and sex. PMI = postmortem interval.

Supplementary Figure 3. NK and CD4 T cell proportions in the brains and other CD8 T cell markers.

(A) Box plot with Y-axis showing differential cell proportions of either NK cells or CD4 T cells among Micro_PVM total cell counts per sample, with age, sex, and PMI being regressed using linear regression. *p*-value are from a regression model including cognitive status, age, sex, and PMI. Multiple testing correction was performed between NK cells and CD4 T cells with Benjamin-Hochberg method (NK cells: 113 cells, CD4 T cells: 82 cells from 84 samples).
(B) Heatmap of subcluster-specific scaled gene expression of selected reference markers of T cells and NK cells populations (see Supplementary Table 1 for associated references for each marker). (579 cells from 78 samples).
(C) UMAP plots showing normalized gene expression of representative markers for the same T cells and NK cells, based on Seurat.

Supplementary Figure 4. Network modules for CD8 T cells in AD brain.

(A) Heatmap of the regression coefficients of the module eigengenes for the network modules, derived from linear regression with mixed random effect models as described in Supplementary Methods. Numbers in the parentheses denote FDR-corrected p-values across the modules. (384 cells from 70 samples)
(B) Box plot of mean module eigengenes per sample between APOE4 carriers and non-carriers for CD8T2 and CD8T4 modules (n=70). *p*-values are from Wilcoxon rank sum test without multiple testing correction.
(C) CD8T2 module network modules with top 20 hub genes. Inner 10 genes of the network plot are top 10 hub genes for CD8T4 module, and the point size denotes kME.

Supplementary Figure 5. Network modules for microglia in AD brain.

(A) Heatmap of the regression coefficients of the module eigengenes for the network modules, derived from linear regression with mixed random effect models as described in Supplementary Methods. Numbers in the parentheses denote FDR-corrected p-values across the modules.

(B) Box plot of mean module eigengenes per sample between APOE4 carriers and non-carriers for mg1 and mg3 modules (n=28). *p*-values are derived from a linear regression model including cognitive status, age, sex, and PMI.

Supplementary Figure 6. Microglial states in AD brain.

UMAP plot of microglial states within Microglia-PVM Seurat object, labeled for ARM, Pathology-associated microglia, Transition microglia, Homeostatic microglia, and Dystrophic microglia. “microglia” represents microglia without specific cell state labels.

Supplementary Figure 7. Absence of correlation between the proportions of homeostatic/dystrophic microglia and CD8 T cells.

(A) Left – Heatmap of odds ratio (module genes vs published markers (Nguyen, A.T. et al., 2020; Smith A. M. et al., 2022) for the group as shown in the x-axis) derived from overrepresentation analysis with Fisher’s exact test. *; $p < 0.05$, **; $p < 0.01$, ***; $p < 0.001$. Right – Numbers of microglial cells labeled as ARM, Pathology-associated (Path), Dystrophic, and Transition microglia.

(B) Scatter plot of cell proportions among total cell counts per sample, with Y-axis showing CD8 T cell proportions (in percentage) and X-axis showing the cell proportions for each microglial state. Colored lines represent regression lines with the slope corresponding with R, derived with *p*-values from Pearson’s correlation coefficients.

Supplementary Figure 8. Cell type abundance per sample in the CCI input.

Number of cells for each cell type per sample based on Multinichenetr analysis. Red horizontal line denotes minimal cell count (5) to be included in the cell-cell interaction analysis. X-axis labels – blue Donor ID represent male patients; red Donor ID are female patients. Italic font Donor ID with underline is APOE4 carriers.

Supplementary Figure 9. Top 50 dementia-specific LR pairs.

Row names denote each ligand-receptor pairs, followed by sender-receiver cell type pairs. The Dot plot represent the products of scaled expressions of ligands and receptors in aggregated expression (pseudobulk)s for each ligand-receptor pair and sample. Heatmap denotes the scaled ligand activity in receiver cell type, which represents the predictability of target gene expressions in the receiver cell type by the ligand based on Multinichenetr analysis.

Supplementary Figure 10. Top 50 dementia-specific LR pairs involving ARM microglia.

Row names denote each ligand-receptor pairs, followed by sender-receiver cell type pairs. The Dot plot represent the products of scaled expressions of ligands and receptors in aggregated expression (pseudobulk)s for each ligand-receptor pair and sample. Heatmap denotes the scaled ligand activity in receiver cell type, which represents the predictability of target gene expressions in the receiver cell type by the ligand based on Multinichenetr analysis.

Supplementary Figure 11. Top 50 dementia-specific LR pairs involving pathology-associated microglia.

Row names denote each ligand-receptor pairs, followed by sender-receiver cell type pairs. The Dot plot represent the products of scaled expressions of ligands and receptors in aggregated expression (pseudobulk)s for each ligand-receptor pair and sample. Heatmap denotes the scaled ligand activity in receiver cell type, which represents the predictability of target gene expressions in the receiver cell type by the ligand based on Multinichenetr analysis.

Supplementary Figure 12. Top 50 dementia-specific LR pairs involving homeostatic microglia.

Row names denote each ligand-receptor pairs, followed by sender-receiver cell type pairs. The Dot plot represent the products of scaled expressions of ligands and receptors in aggregated expression (pseudobulk) for each ligand-receptor pair and sample. Heatmap denotes the scaled ligand activity in receiver cell type, which represents the predictability of target gene expressions in the receiver cell type by the ligand based on Multinichenetr analysis.

Supplementary Figure 13. Top 50 dementia-specific LR pairs involving dystrophic microglia.

Row names denote each ligand-receptor pairs, followed by sender-receiver cell type pairs. The Dot plot represent the products of scaled expressions of ligands and receptors in aggregated expression (pseudobulk) for each ligand-receptor pair and sample. Heatmap denotes the scaled ligand activity in receiver cell type, which represents the predictability of target gene expressions in the receiver cell type by the ligand based on Multinichenetr analysis.

Supplementary Figure 14. Top 50 dementia-specific LR pairs between CD8 T cells and microglia.

Row names denote each ligand-receptor pairs, followed by sender-receiver cell type pairs. The Dot plot represent the products of scaled expressions of ligands and receptors in aggregated expression (pseudobulk) for each ligand-receptor pair and sample. Heatmap denotes the scaled ligand activity in receiver cell type, which represents the predictability of target gene expressions in the receiver cell type by the ligand based on Multinichenetr analysis.

Supplementary Figure 15. Top 50 dementia-specific LR pairs between CD8 T cells and neurons.

Row names denote each ligand-receptor pairs, followed by sender-receiver cell type pairs. The Dot plot represent the products of scaled expressions of ligands and receptors in aggregated expression (pseudobulk) for each ligand-receptor pair and sample. The middle heatmap denotes the scaled ligand activity in receiver cell type, which represents the predictability of target gene expressions in the receiver cell type by the ligand based on Multinichenetr analysis. The right heatmap denotes network connectivity (kME) of each gene for the ligand or receptor on CD8 T cells for CD8T2 (cytotoxic) or CD8T4 (immunomodulatory) modules derived from WGCNA.

Supplementary Figure 16. Differential expression of NOTCH2 in ARM across APOE4 carrier status.

Violin plot with overlaying box plots of normalized NOTCH2 expression among ARM. ARM module was projected to microglia from all samples (n=84) and then extracted as top 20 % ranked by the module eigengene, similar to the subsampled microglia described in the manuscript. *p*-values are derived from linear regression with mixed random effect models, including APOE4 carrier status, age, sex, and PMI; with donor ID as the random effect. Multiple testing correction was not performed.
