# Supplementary material for: Cell States and Interactions of CD8 T Cells and Disease-Enriched Microglia in Human Brains with Alzheimer’s Disease
Source: Biomedicines. 2024 Jan 25;12(2):308. doi: 10.3390/biomedicines12020308 (PMC10886701; doi:10.3390/biomedicines12020308)
Supplement: Supplementary file 1 [file biomedicines-12-00308-s001.zip › Supplementary Methods.pdf]

Supplementary Methods

1. Clustering and identification of CD8 T cells within the microglial cluster

    Normalization and clustering was performed using Seurat NormalizeData() and FindNeighbors()/FindClusters() functions on the Microglia-PVM object with the following parameters: dims = 1:40, resolution = 1.2. The CD247 (+) lymphocytic clusters (cluster 19 and 37) were subjected to repeat clustering with following parameters: dims = 1:20, resolution = 1.2.

2. Proportions of lymphocytes among microglial cluster and correlation analysis using multivariable linear regressions

    Cell proportions of lymphocytes among microglial cluster per library were calculated with lymphocytic cluster counts divided by the total cell counts of the library (donor) in the Microglia_PVM Seurat object. Multivariable linear regression was performed with the following formula:

    Log2(Cell proportions) ~ Disease + age + sex + post mortem interval

    We also performed correlation analysis substituting Disease in the above formula with AD pathological stages (ADNC, Thal phase, CERAD score, and Braak stages) in dementia cases, treating each staging as numerical interval variables to derive covariables-controlled *p*-values for each comparison.

3. Differential gene expression analysis of CD8 T cells

    Aggregated expression count matrix of CD8 T cells per donor was calculated using Seurat AggregateExpression() function. Then this aggregated expression matrix was subjected to differential gene expression analysis using limma-voom as described before [1]. Genes were filtered to achieve optimal mean-variance curve without near-0 inflation of the standard deviation (cutoff counts = 200).

4. hdWGCNA of microglia and CD8 T cells

    For microglia, hdWGCNA was performed with default parameters with soft_power = 6, and metacells generation was specified to be grouped by donor ID. For CD8 T cells, hdWGCNA was performed with following parameters: soft_power = 16, corType = 'bicor'. minModuleSize = 100, mergeCutHeight = 0.3. Module-trait correlation was assessed using lme4 package with the following mixed random effect model:

    harmonized module eigengene ~ 1 + disease + post mortem interval + age + sex + (1 | donor_id)

    p-values for each coefficient are corrected across the models for each module using Benjamini-Hochberg method.

5. Cell-cell interaction analysis using Multinichenetr

    All major cell types and annotated microglia and CD8 T cells were included as the input for CCI analysis using Multinichenet, with all default parameters except for minimum cells = 5 per cell type (default: 10) and ligand or receptor specificity weights = 3 (default: 2).

Reference

1. Squair, J.W.; Gautier, M.; Kathe, C.; Anderson, M.A.; James, N.D.; Hutson, T.H.; Hudelle, R.; Qaiser, T.; Matson, K.J.E.; Barraud, Q.; et al. Confronting False Discoveries in Single-Cell Differential Expression. Nature Communications 2021 12:1 2021, 12, 1–15, doi:10.1038/s41467-021-25960-2.
