# Supplementary material for: Cell States and Interactions of CD8 T Cells and Disease-Enriched Microglia in Human Brains with Alzheimer’s Disease
Source: Biomedicines. 2024 Jan 25;12(2):308. doi: 10.3390/biomedicines12020308 (PMC10886701; doi:10.3390/biomedicines12020308)
Supplement: Supplementary file 1 [file biomedicines-12-00308-s001.zip › Supplementary_Figure_S10.pdf]

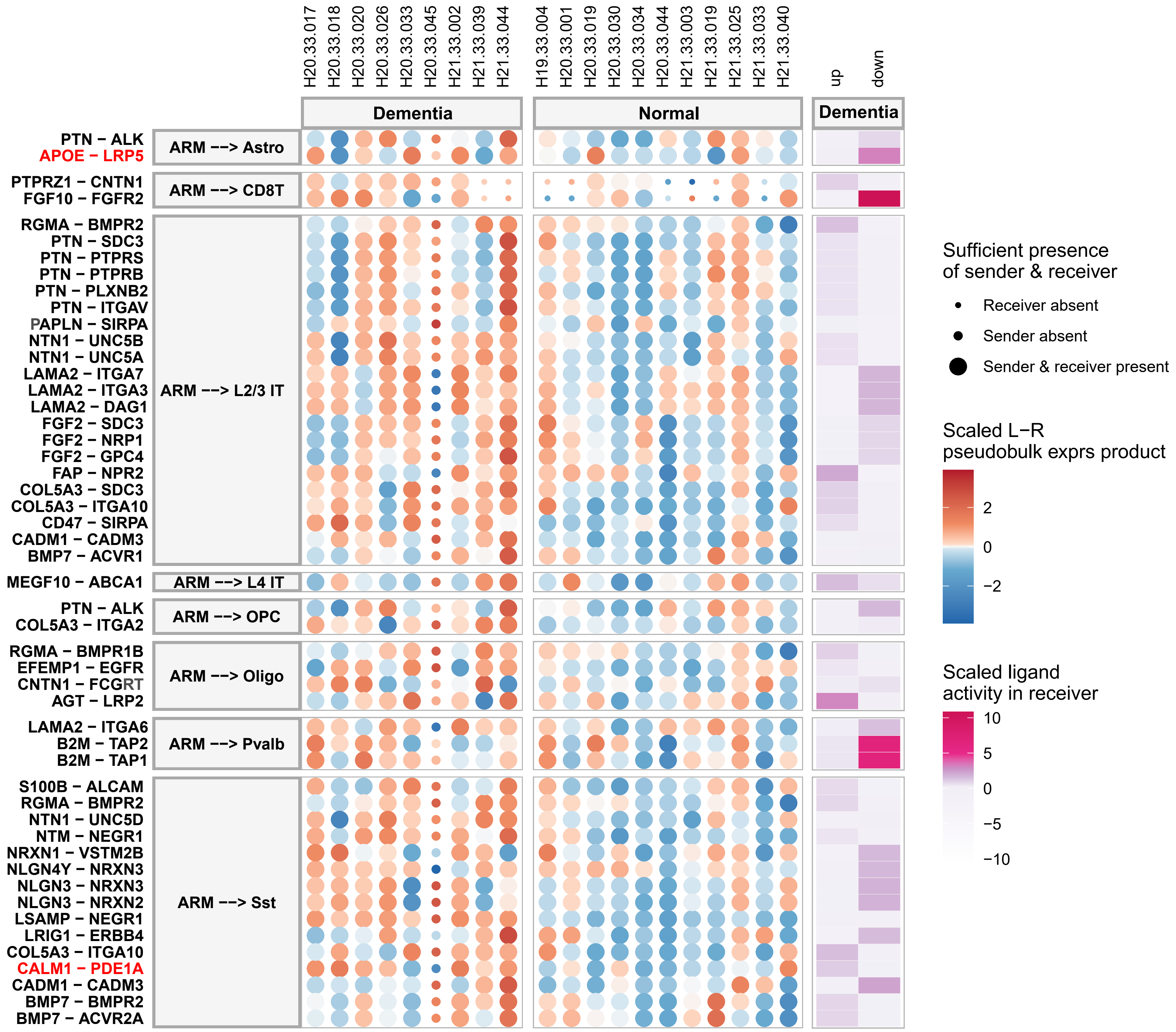

H20.33.017
H20.33.018
H20.33.020
H20.33.026
H20.33.033
H20.33.045
H21.33.002
H21.33.039
H21.33.044
H19.33.004
H20.33.001
H20.33.019
H20.33.030
H20.33.034
H20.33.044
H21.33.003
H21.33.019
H21.33.025
H21.33.033
H21.33.040
up
down
Dementia
Normal
Dementia
PTN − ALK
APOE − LRP5
ARM --> Astro
PTPRZ1 − CNTN1
FGF10 − FGFR2
ARM --> CD8T
RGMA − BMPR2
PTN − SDC3
PTN − PTPRS
PTN − PTPRB
PTN − PLXNB2
PTN − ITGAV
PAPLN − SIRPA
NTN1 − UNC5B
NTN1 − UNC5A
LAMA2 − ITGA7
LAMA2 − ITGA3
LAMA2 − DAG1
FGF2 − SDC3
FGF2 − NRP1
FGF2 − GPC4
FAP − NPR2
COL5A3 − SDC3
COL5A3 − ITGA10
CD47 − SIRPA
CADM1 − CADM3
BMP7 − ACVR1
ARM --> L2/3 IT
MEGF10 − ABCA1
ARM --> L4 IT
PTN − ALK
COL5A3 − ITGA2
ARM --> OPC
RGMA − BMPR1B
EFEMP1 − EGFR
CNTN1 − FCGRT
AGT − LRP2
ARM --> Oligo
LAMA2 − ITGA6
B2M − TAP2
B2M − TAP1
ARM --> Pvalb
S100B − ALCAM
RGMA − BMPR2
NTN1 − UNC5D
NTM − NEGR1
NRXN1 − VSTM2B
NLGN4Y − NRXN3
NLGN3 − NRXN3
NLGN3 − NRXN2
LSAMP − NEGR1
LRIG1 − ERBB4
COL5A3 − ITGA10
CALM1 − PDE1A
CADM1 − CADM3
BMP7 − BMPR2
BMP7 − ACVR2A
ARM --> Sst
Sufficient presence of sender & receiver
Receiver absent
Sender absent
Sender & receiver present
Scaled L−R pseudobulk exprs product
2
0
−2
Scaled ligand activity in receiver
10
5
0
−5
−10

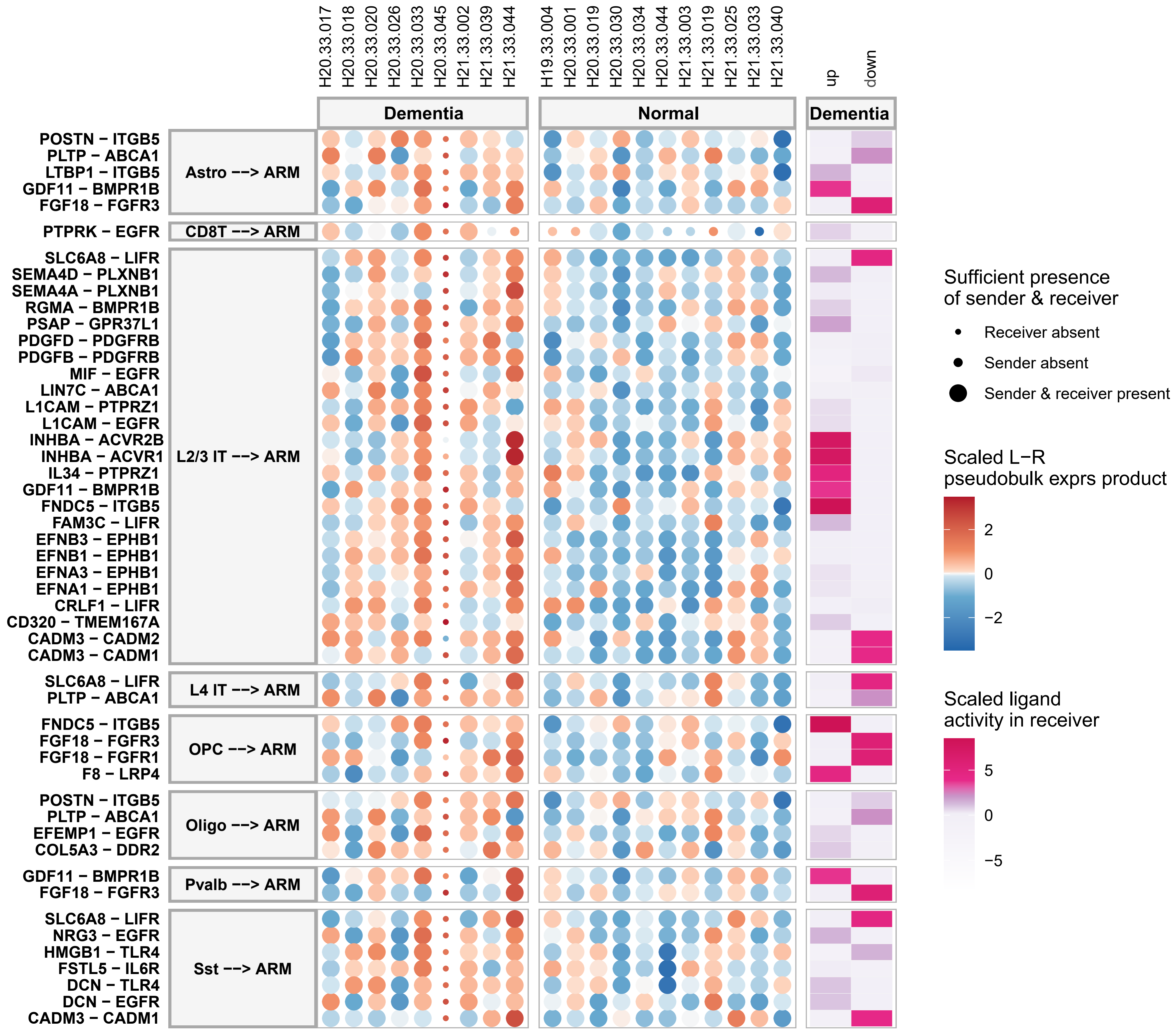

H20.33.017
H20.33.018
H20.33.020
H20.33.026
H20.33.033
H20.33.045
H21.33.002
H21.33.039
H21.33.044
H19.33.004
H20.33.001
H20.33.019
H20.33.030
H20.33.034
H20.33.044
H21.33.003
H21.33.019
H21.33.025
H21.33.033
H21.33.040
up
down
Dementia
Normal
Dementia
POSTN − ITGB5
PLTP − ABCA1
LTBP1 − ITGB5
GDF11 − BMPR1B
FGF18 − FGFR3
Astro --> ARM
PTPRK − EGFR
CD8T --> ARM
SLC6A8 − LIFR
SEMA4D − PLXNB1
SEMA4A − PLXNB1
RGMA − BMPR1B
PSAP − GPR37L1
PDGFD − PDGFRB
PDGFB − PDGFRB
MIF − EGFR
LIN7C − ABCA1
L1CAM − PTPRZ1
L1CAM − EGFR
INHBA − ACVR2B
INHBA − ACVR1
IL34 − PTPRZ1
GDF11 − BMPR1B
FNDC5 − ITGB5
FAM3C − LIFR
EFNB3 − EPHB1
EFNB1 − EPHB1
EFNA3 − EPHB1
EFNA1 − EPHB1
CRLF1 − LIFR
CD320 − TMEM167A
CADM3 − CADM2
CADM3 − CADM1
L2/3 IT --> ARM
SLC6A8 − LIFR
PLTP − ABCA1
L4 IT --> ARM
FNDC5 − ITGB5
FGF18 − FGFR3
FGF18 − FGFR1
F8 − LRP4
OPC --> ARM
POSTN − ITGB5
PLTP − ABCA1
EFEMP1 − EGFR
COL5A3 − DDR2
Oligo --> ARM
GDF11 − BMPR1B
FGF18 − FGFR3
Pvalb --> ARM
SLC6A8 − LIFR
NRG3 − EGFR
HMGB1 − TLR4
FSTL5 − IL6R
DCN − TLR4
DCN − EGFR
CADM3 − CADM1
Sst --> ARM
Sufficient presence of sender & receiver
Receiver absent
Sender absent
Sender & receiver present
Scaled L−R pseudobulk exprs product
2
0
−2
Scaled ligand activity in receiver
5
0
−5
