# Supplementary material for: Cell States and Interactions of CD8 T Cells and Disease-Enriched Microglia in Human Brains with Alzheimer’s Disease
Source: Biomedicines. 2024 Jan 25;12(2):308. doi: 10.3390/biomedicines12020308 (PMC10886701; doi:10.3390/biomedicines12020308)
Supplement: Supplementary file 1 [file biomedicines-12-00308-s001.zip › Supplementary_Figure_S12.pdf]

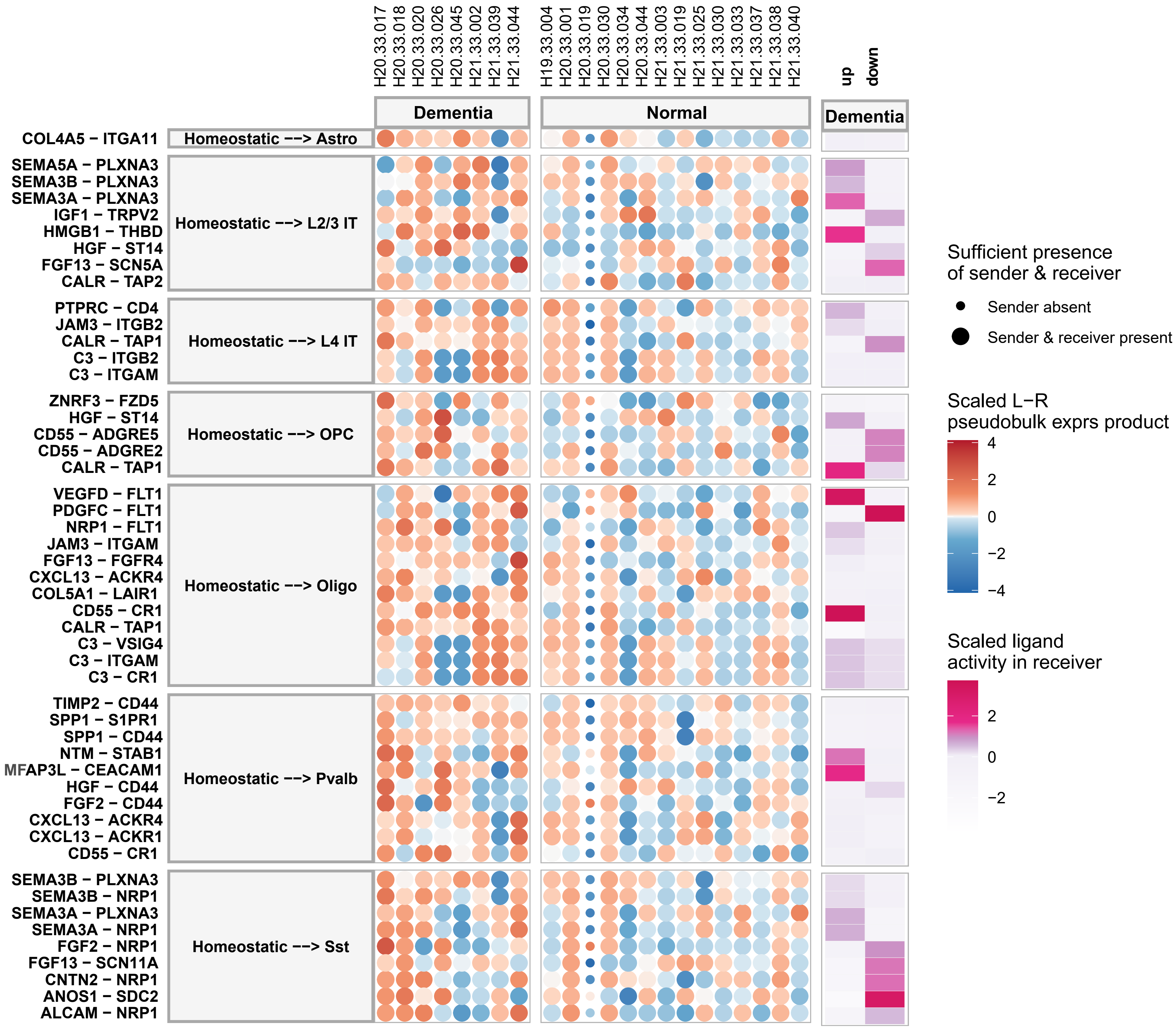

H20.33.017
H20.33.018
H20.33.020
H20.33.026
H20.33.045
H21.33.002
H21.33.039
H21.33.044
H19.33.004
H20.33.001
H20.33.019
H20.33.030
H20.33.034
H20.33.044
H21.33.003
H21.33.019
H21.33.025
H21.33.030
H21.33.033
H21.33.037
H21.33.038
H21.33.040
up
down
Dementia
Normal
Dementia
COL4A5 − ITGA11
Homeostatic --> Astro
SEMA5A − PLXNA3
SEMA3B − PLXNA3
SEMA3A − PLXNA3
IGF1 − TRPV2
HMGB1 − THBD
HGF − ST14
FGF13 − SCN5A
CALR − TAP2
Homeostatic --> L2/3 IT
PTPRC − CD4
JAM3 − ITGB2
CALR − TAP1
C3 − ITGB2
C3 − ITGAM
Homeostatic --> L4 IT
ZNRF3 − FZD5
HGF − ST14
CD55 − ADGRE5
CD55 − ADGRE2
CALR − TAP1
Homeostatic --> OPC
VEGFD − FLT1
PDGFC − FLT1
NRP1 − FLT1
JAM3 − ITGAM
FGF13 − FGFR4
CXCL13 − ACKR4
COL5A1 − LAIR1
CD55 − CR1
CALR − TAP1
C3 − VSIG4
C3 − ITGAM
C3 − CR1
Homeostatic --> Oligo
TIMP2 − CD44
SPP1 − S1PR1
SPP1 − CD44
NTM − STAB1
MFAP3L − CEACAM1
HGF − CD44
FGF2 − CD44
CXCL13 − ACKR4
CXCL13 − ACKR1
CD55 − CR1
Homeostatic --> Pvalb
SEMA3B − PLXNA3
SEMA3B − NRP1
SEMA3A − PLXNA3
SEMA3A − NRP1
FGF2 − NRP1
FGF13 − SCN11A
CNTN2 − NRP1
ANOS1 − SDC2
ALCAM − NRP1
Homeostatic --> Sst
Sufficient presence of sender & receiver
Sender absent
Sender & receiver present
Scaled L−R pseudobulk exprs product
4
2
0
−2
−4
Scaled ligand activity in receiver
2
0
−2

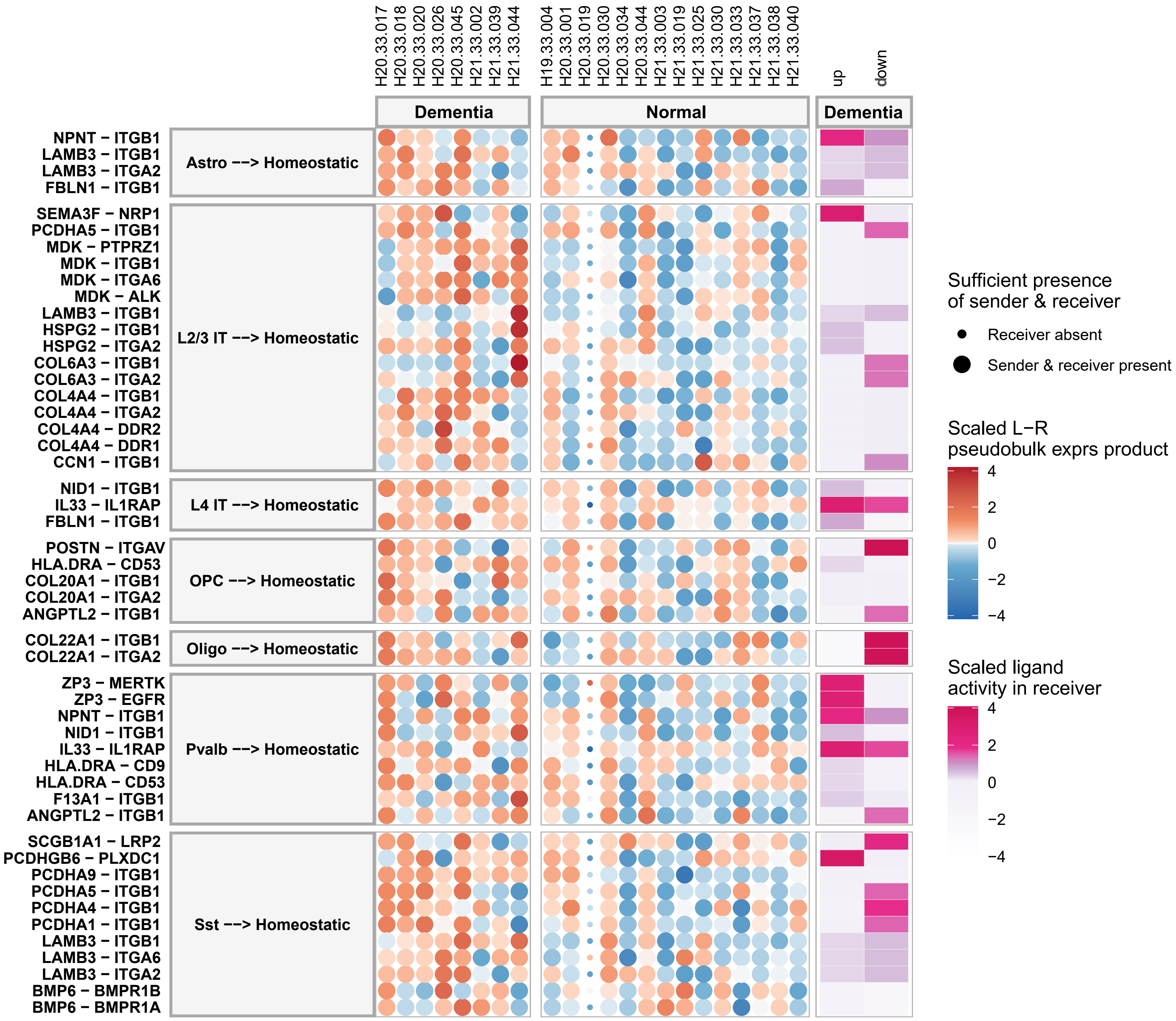
H20.33.017
H20.33.018
H20.33.020
H20.33.026
H20.33.045
H21.33.002
H21.33.039
H21.33.044
H19.33.004
H20.33.001
H20.33.019
H20.33.030
H20.33.034
H20.33.044
H21.33.003
H21.33.019
H21.33.025
H21.33.030
H21.33.033
H21.33.037
H21.33.038
H21.33.040
up
down
Dementia
Normal
Dementia
Astro --> Homeostatic
NPNT − ITGB1
LAMB3 − ITGB1
LAMB3 − ITGA2
FBLN1 − ITGB1
L2/3 IT --> Homeostatic
SEMA3F − NRP1
PCDHA5 − ITGB1
MDK − PTPRZ1
MDK − ITGB1
MDK − ITGA6
MDK − ALK
LAMB3 − ITGB1
HSPG2 − ITGB1
HSPG2 − ITGA2
COL6A3 − ITGB1
COL6A3 − ITGA2
COL4A4 − ITGB1
COL4A4 − ITGA2
COL4A4 − DDR2
COL4A4 − DDR1
CCN1 − ITGB1
L4 IT --> Homeostatic
NID1 − ITGB1
IL33 − IL1RAP
FBLN1 − ITGB1
OPC --> Homeostatic
POSTN − ITGAV
HLA.DRA − CD53
COL20A1 − ITGB1
COL20A1 − ITGA2
ANGPTL2 − ITGB1
Oligo --> Homeostatic
COL22A1 − ITGB1
COL22A1 − ITGA2
Pvalb --> Homeostatic
ZP3 − MERTK
ZP3 − EGFR
NPNT − ITGB1
NID1 − ITGB1
IL33 − IL1RAP
HLA.DRA − CD9
HLA.DRA − CD53
F13A1 − ITGB1
ANGPTL2 − ITGB1
Sst --> Homeostatic
SCGB1A1 − LRP2
PCDHGB6 − PLXDC1
PCDHA9 − ITGB1
PCDHA5 − ITGB1
PCDHA4 − ITGB1
PCDHA1 − ITGB1
LAMB3 − ITGB1
LAMB3 − ITGA6
LAMB3 − ITGA2
BMP6 − BMPR1B
BMP6 − BMPR1A
Sufficient presence of sender & receiver
Receiver absent
Sender & receiver present
Scaled L−R pseudobulk exprs product
4
2
0
−2
−4
Scaled ligand activity in receiver
4
2
0
−2
−4
