# Supplementary material for: Cell States and Interactions of CD8 T Cells and Disease-Enriched Microglia in Human Brains with Alzheimer’s Disease
Source: Biomedicines. 2024 Jan 25;12(2):308. doi: 10.3390/biomedicines12020308 (PMC10886701; doi:10.3390/biomedicines12020308)
Supplement: Supplementary file 1 [file biomedicines-12-00308-s001.zip › Supplementary_Figure_S13.pdf]

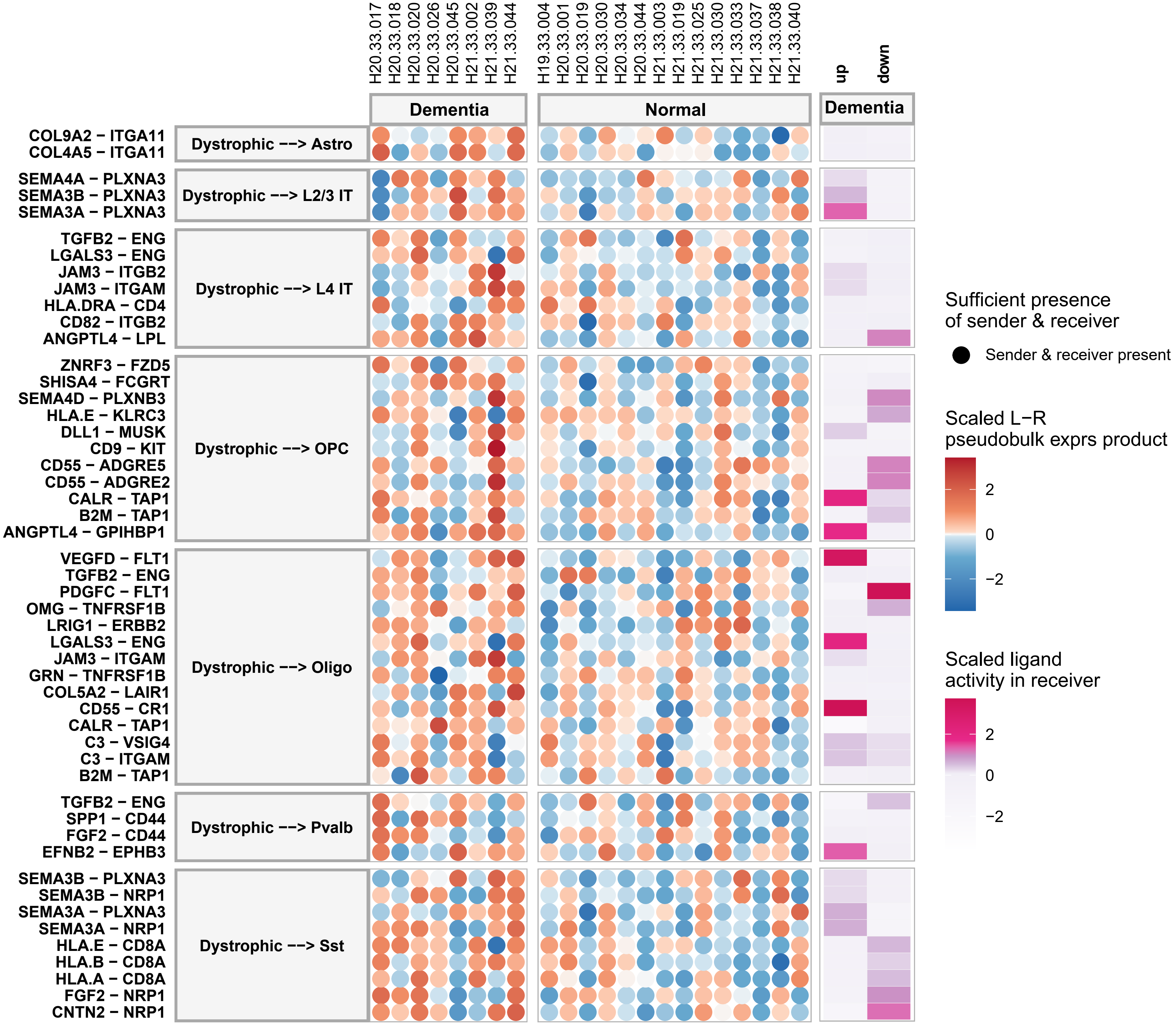

H20.33.017
H20.33.018
H20.33.020
H20.33.026
H20.33.045
H21.33.002
H21.33.039
H21.33.044
H19.33.004
H20.33.001
H20.33.019
H20.33.030
H20.33.034
H20.33.044
H21.33.003
H21.33.019
H21.33.025
H21.33.030
H21.33.033
H21.33.037
H21.33.038
H21.33.040
up
down
Dementia
Normal
Dementia
COL9A2 − ITGA11
COL4A5 − ITGA11
Dystrophic --> Astro
SEMA4A − PLXNA3
SEMA3B − PLXNA3
SEMA3A − PLXNA3
Dystrophic --> L2/3 IT
TGFB2 − ENG
LGALS3 − ENG
JAM3 − ITGB2
JAM3 − ITGAM
HLA.DRA − CD4
CD82 − ITGB2
ANGPTL4 − LPL
Dystrophic --> L4 IT
ZNRF3 − FZD5
SHISA4 − FCGRT
SEMA4D − PLXNB3
HLA.E − KLRC3
DLL1 − MUSK
CD9 − KIT
CD55 − ADGRE5
CD55 − ADGRE2
CALR − TAP1
B2M − TAP1
ANGPTL4 − GPIHBP1
Dystrophic --> OPC
VEGFD − FLT1
TGFB2 − ENG
PDGFC − FLT1
OMG − TNFRSF1B
LRIG1 − ERBB2
LGALS3 − ENG
JAM3 − ITGAM
GRN − TNFRSF1B
COL5A2 − LAIR1
CD55 − CR1
CALR − TAP1
C3 − VSIG4
C3 − ITGAM
B2M − TAP1
Dystrophic --> Oligo
TGFB2 − ENG
SPP1 − CD44
FGF2 − CD44
EFNB2 − EPHB3
Dystrophic --> Pvalb
SEMA3B − PLXNA3
SEMA3B − NRP1
SEMA3A − PLXNA3
SEMA3A − NRP1
HLA.E − CD8A
HLA.B − CD8A
HLA.A − CD8A
FGF2 − NRP1
CNTN2 − NRP1
Dystrophic --> Sst
Sufficient presence of sender & receiver
Sender & receiver present
Scaled L−R pseudobulk exprs product
2
0
−2
Scaled ligand activity in receiver
2
0
−2

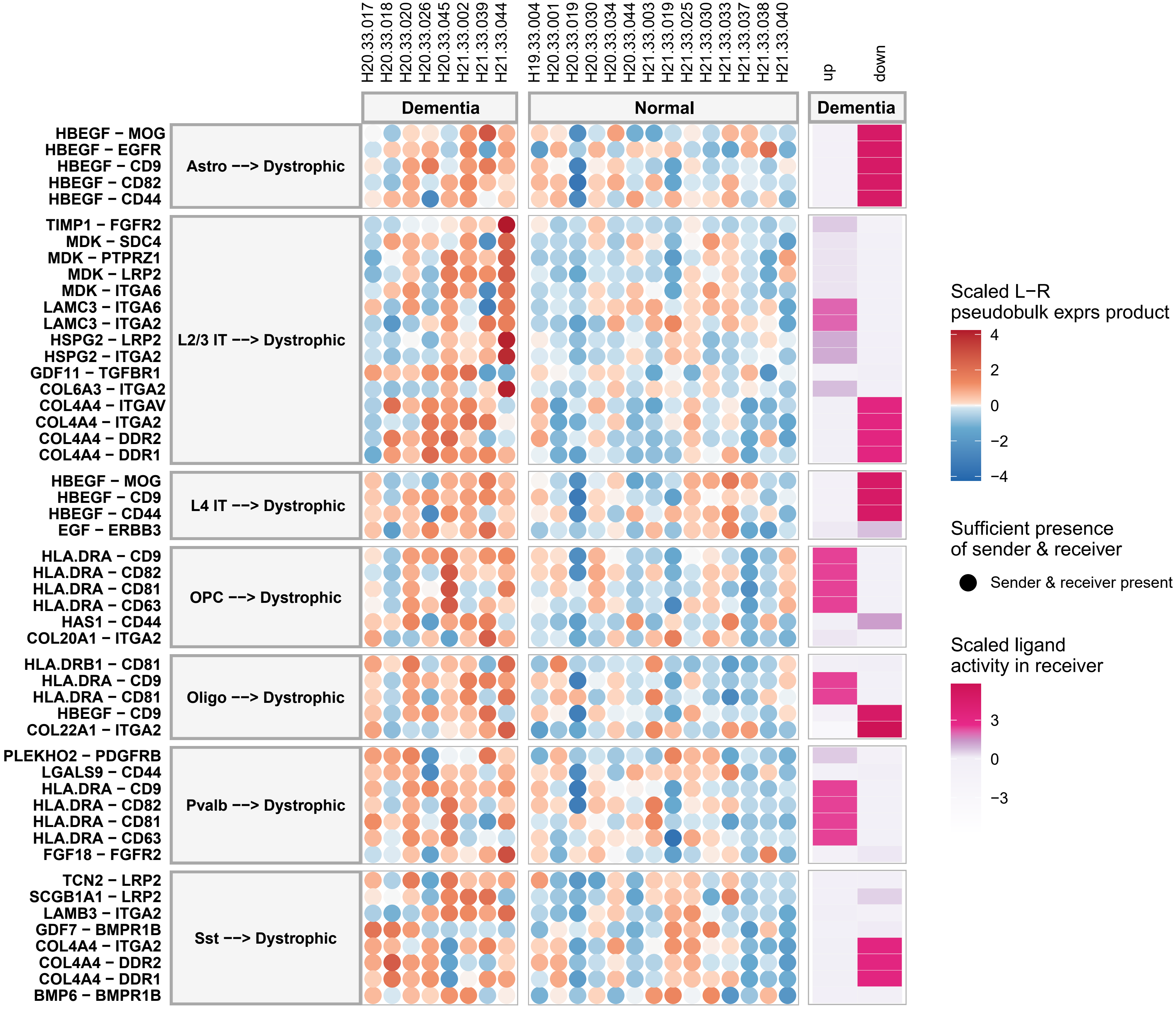

H20.33.017
H20.33.018
H20.33.020
H20.33.026
H20.33.045
H21.33.002
H21.33.039
H21.33.044
H19.33.004
H20.33.001
H20.33.019
H20.33.030
H20.33.034
H20.33.044
H21.33.003
H21.33.019
H21.33.025
H21.33.030
H21.33.033
H21.33.037
H21.33.038
H21.33.040
up
down
Dementia
Normal
Dementia
HBEGF − MOG
HBEGF − EGFR
HBEGF − CD9
HBEGF − CD82
HBEGF − CD44
Astro --> Dystrophic
TIMP1 − FGFR2
MDK − SDC4
MDK − PTPRZ1
MDK − LRP2
MDK − ITGA6
LAMC3 − ITGA6
LAMC3 − ITGA2
HSPG2 − LRP2
HSPG2 − ITGA2
GDF11 − TGFBR1
COL6A3 − ITGA2
COL4A4 − ITGAV
COL4A4 − ITGA2
COL4A4 − DDR2
COL4A4 − DDR1
L2/3 IT --> Dystrophic
HBEGF − MOG
HBEGF − CD9
HBEGF − CD44
EGF − ERBB3
L4 IT --> Dystrophic
HLA.DRA − CD9
HLA.DRA − CD82
HLA.DRA − CD81
HLA.DRA − CD63
HAS1 − CD44
COL20A1 − ITGA2
OPC --> Dystrophic
HLA.DRB1 − CD81
HLA.DRA − CD9
HLA.DRA − CD81
HBEGF − CD9
COL22A1 − ITGA2
Oligo --> Dystrophic
PLEKHO2 − PDGFRB
LGALS9 − CD44
HLA.DRA − CD9
HLA.DRA − CD82
HLA.DRA − CD81
HLA.DRA − CD63
FGF18 − FGFR2
Pvalb --> Dystrophic
TCN2 − LRP2
SCGB1A1 − LRP2
LAMB3 − ITGA2
GDF7 − BMPR1B
COL4A4 − ITGA2
COL4A4 − DDR2
COL4A4 − DDR1
BMP6 − BMPR1B
Sst --> Dystrophic
Scaled L−R
pseudobulk exprs product
4
2
0
−2
−4
Sufficient presence
of sender & receiver
Sender & receiver present
Scaled ligand
activity in receiver
3
0
−3
