# Supplementary material for: Cell States and Interactions of CD8 T Cells and Disease-Enriched Microglia in Human Brains with Alzheimer’s Disease
Source: Biomedicines. 2024 Jan 25;12(2):308. doi: 10.3390/biomedicines12020308 (PMC10886701; doi:10.3390/biomedicines12020308)
Supplement: Supplementary file 1 [file biomedicines-12-00308-s001.zip › Supplementary_Figure_S6.pdf]

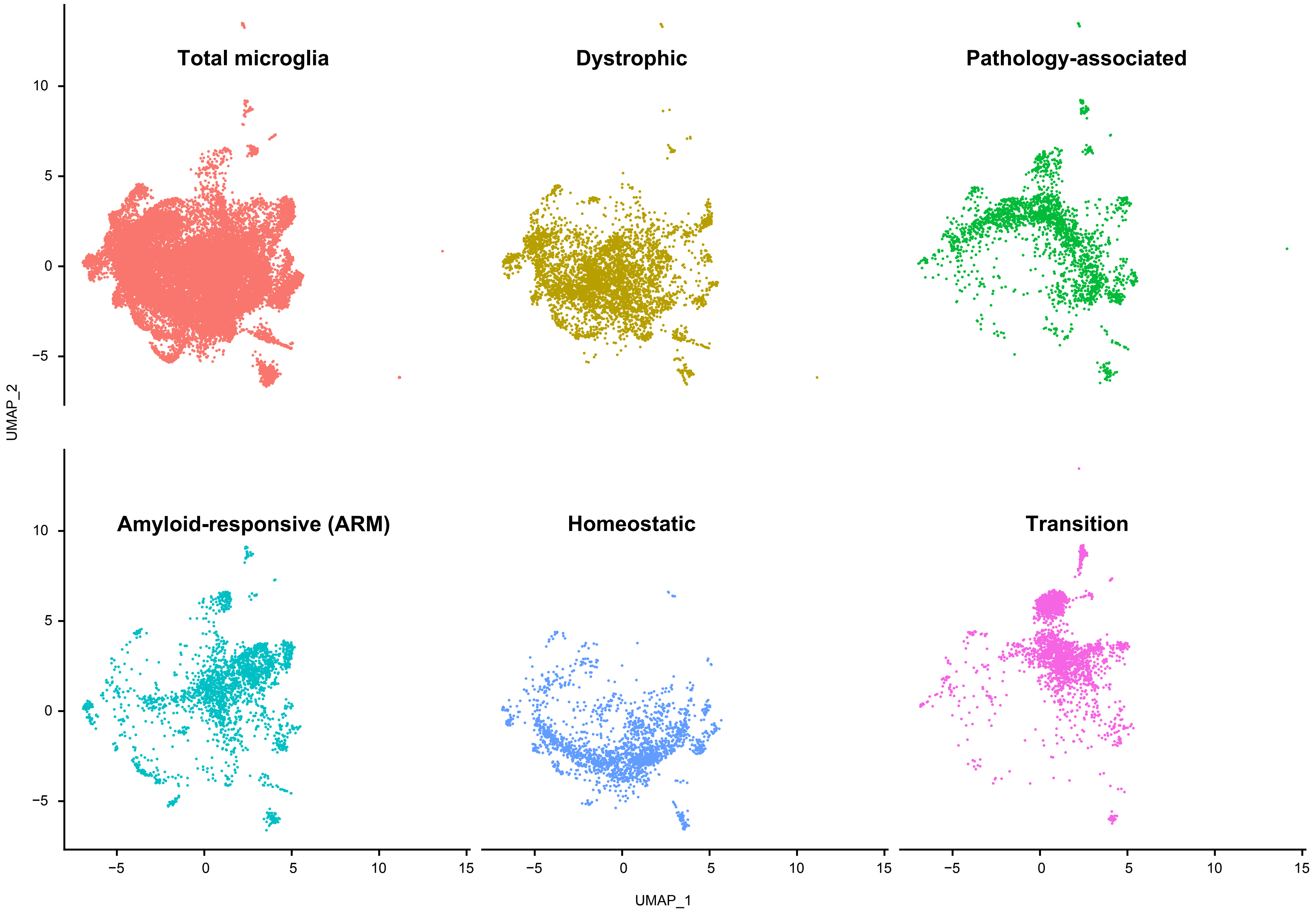
Total microglia
Dystrophic
Pathology-associated
Amyloid-responsive (ARM)
Homeostatic
Transition
UMAP_2
UMAP_1
10
5
0
−5
−5
0
5
10
15
