# Supplementary figures and images for: Cell States and Interactions of CD8 T Cells and Disease-Enriched Microglia in Human Brains with Alzheimer’s Disease
Source: Biomedicines. 2024 Jan 25;12(2):308. doi: 10.3390/biomedicines12020308 (PMC10886701; doi:10.3390/biomedicines12020308)

## Lewy body pathology

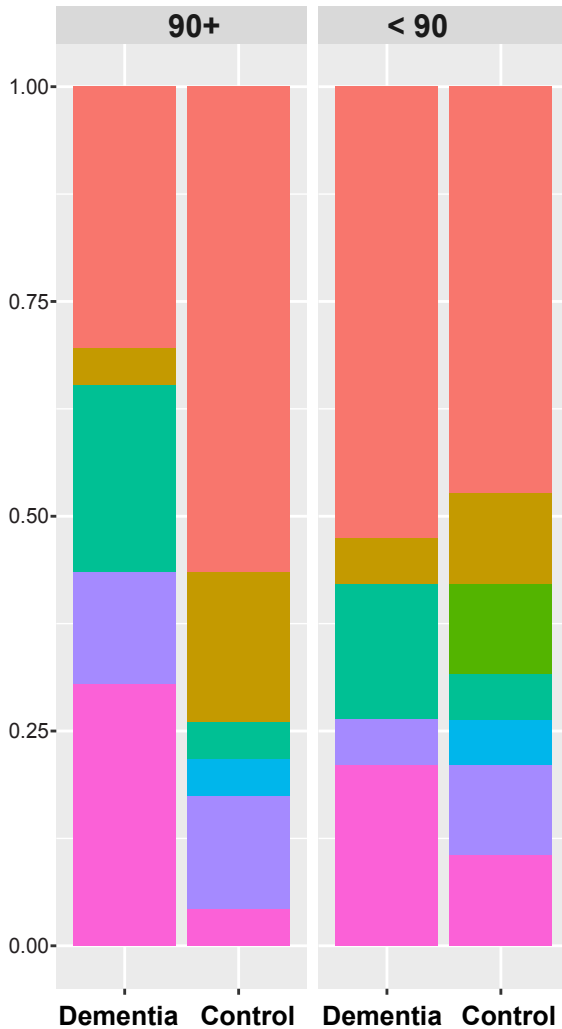

## Lewy body disease pathology

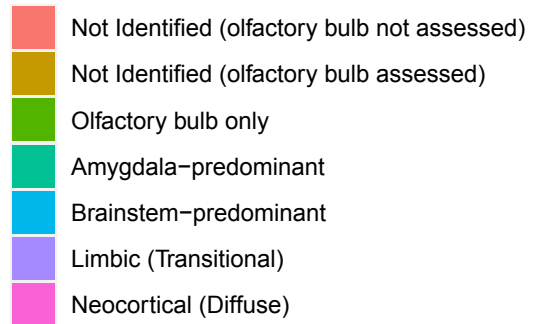

Supplement: Supplementary file 1 [file biomedicines-12-00308-s001.zip › Supplementary_Figure_S1.pdf]

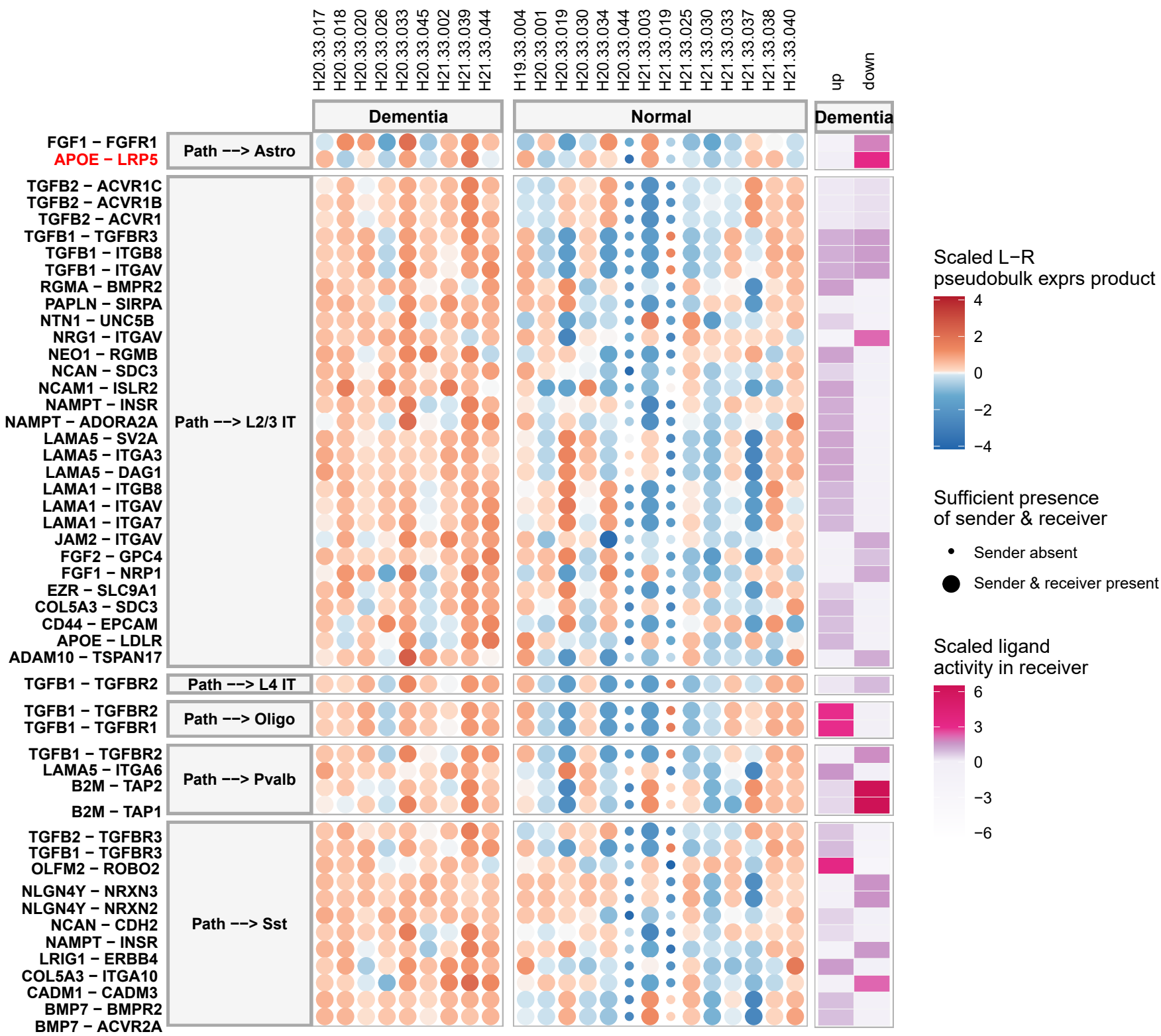

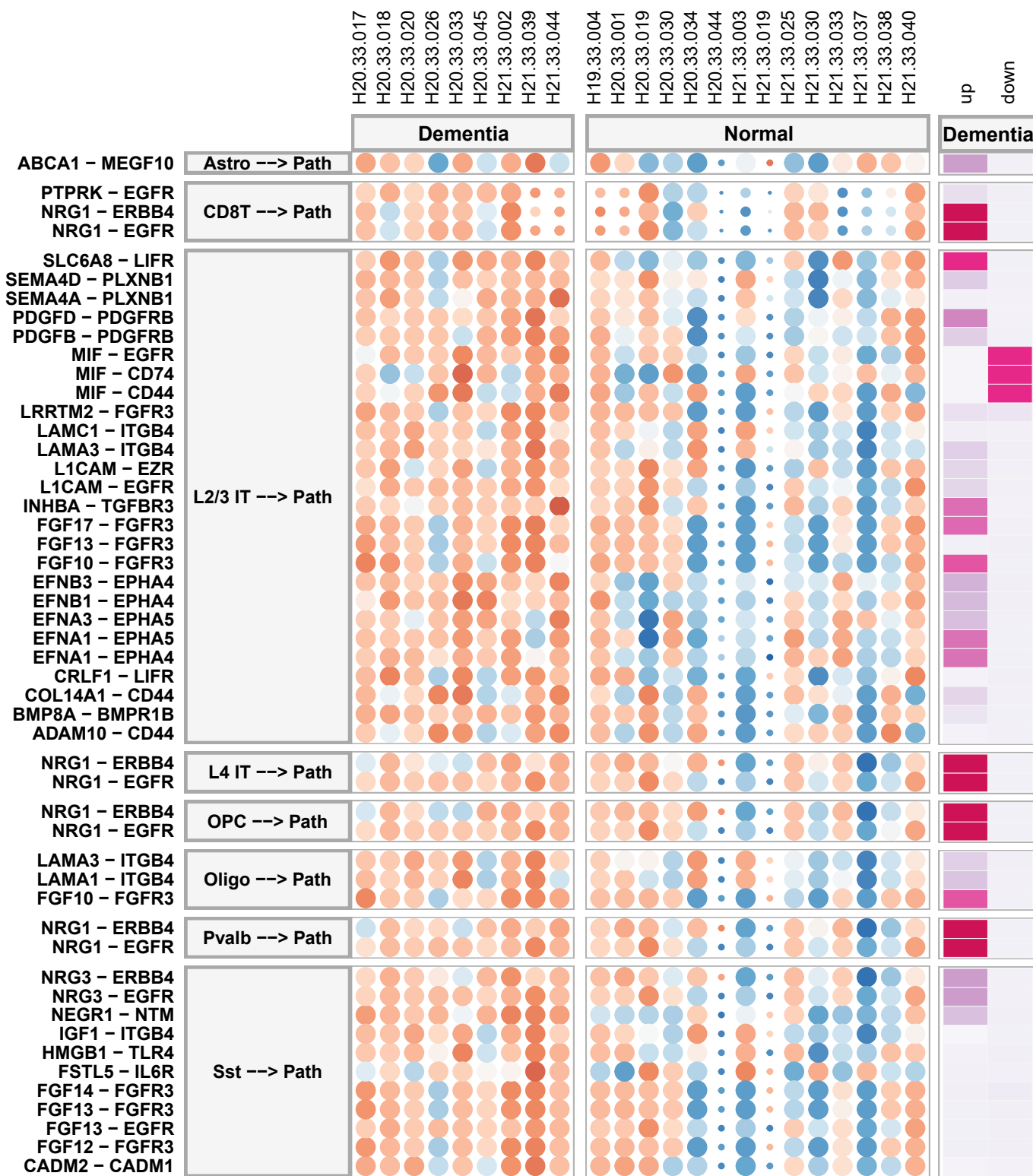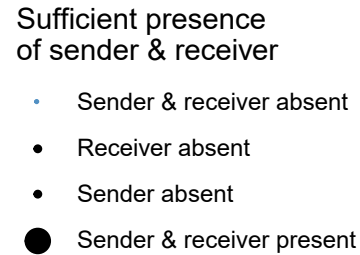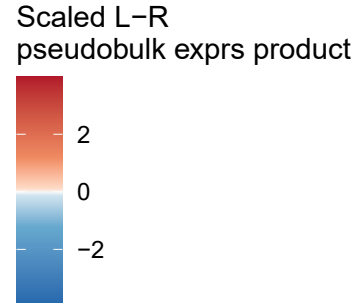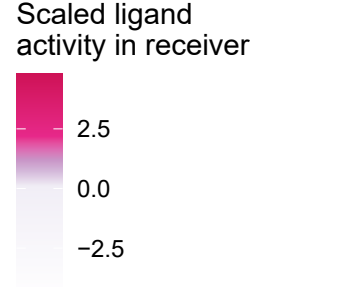

Supplement: Supplementary file 1 [file biomedicines-12-00308-s001.zip › Supplementary_Figure_S11.pdf]

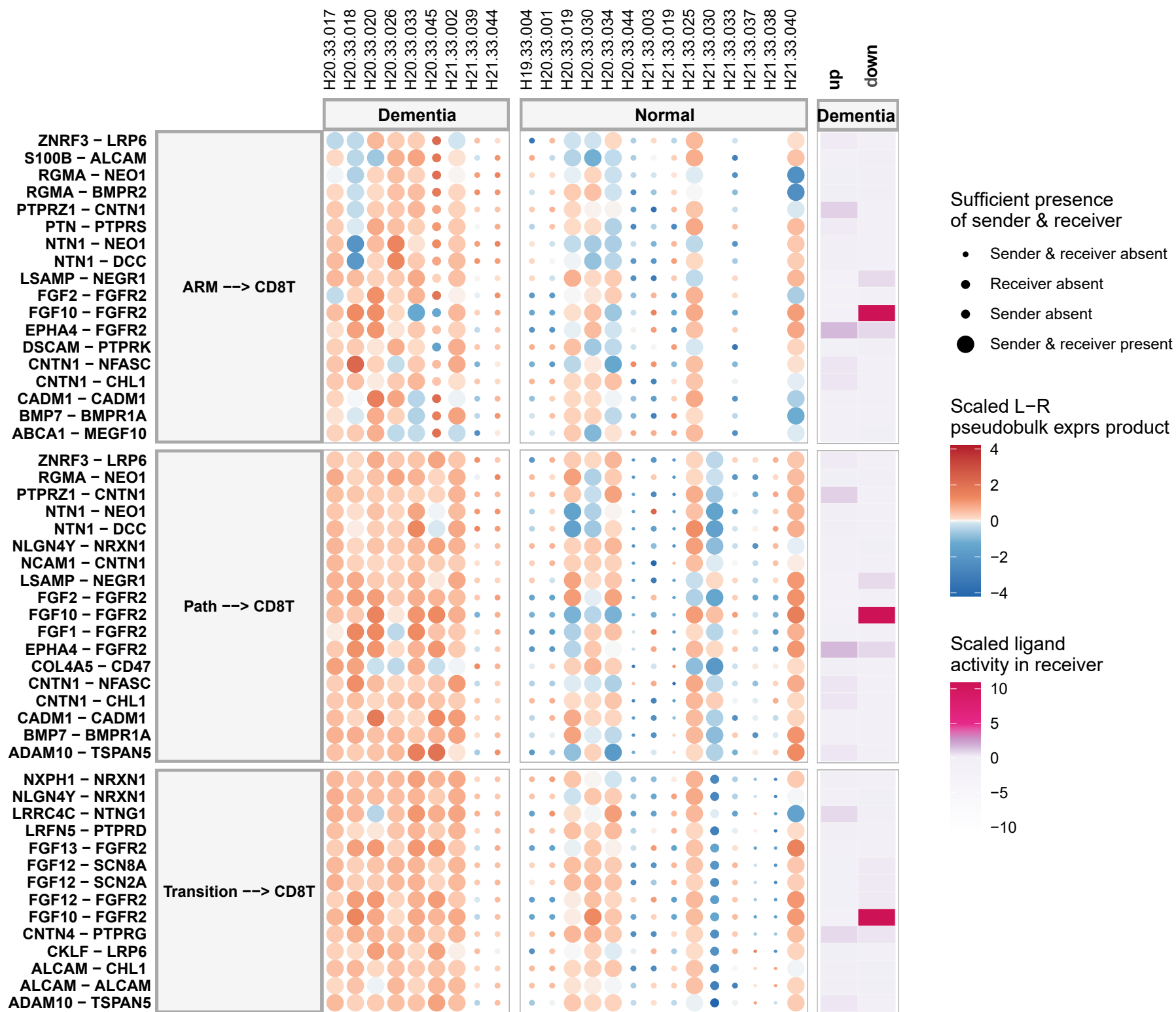

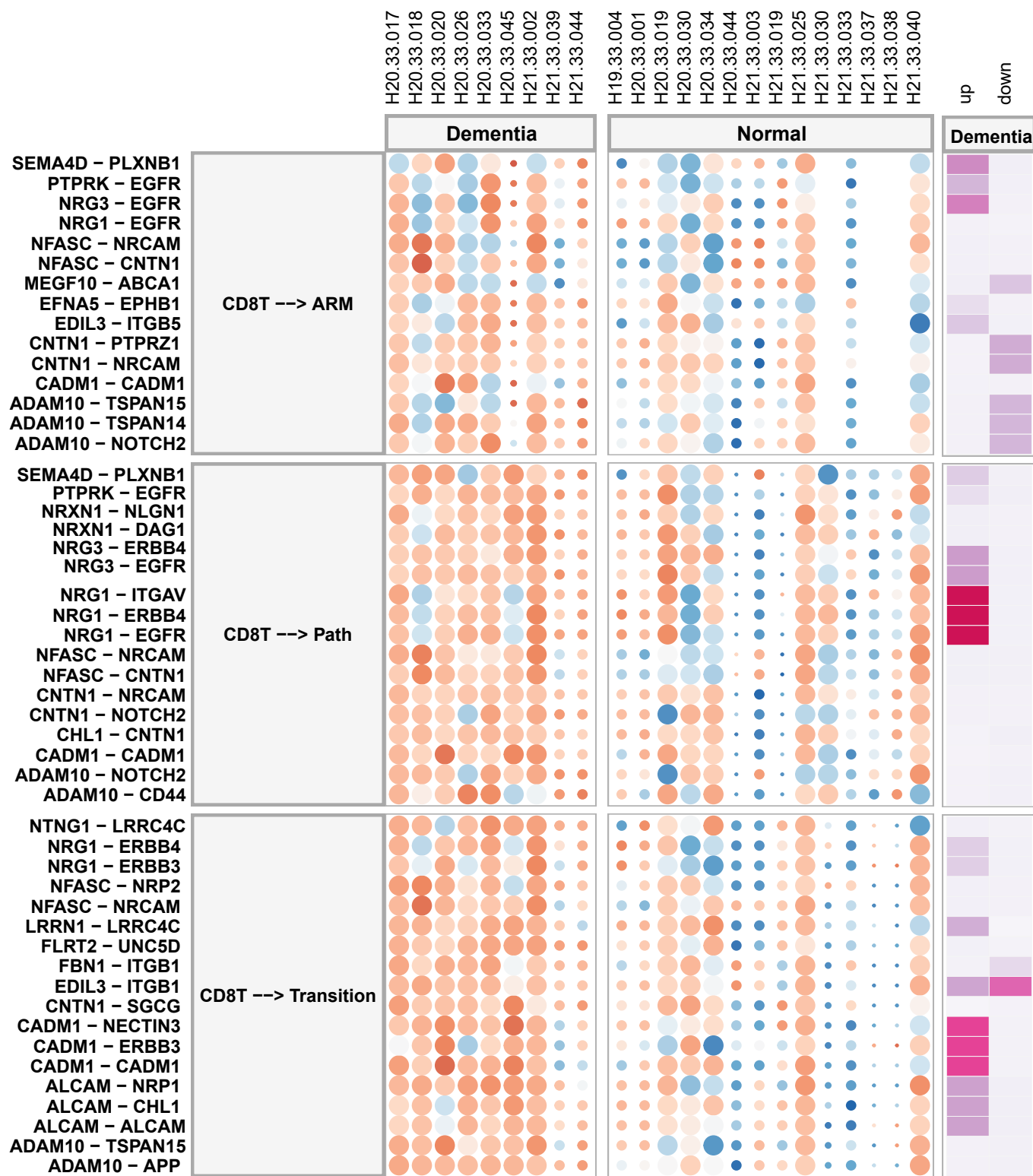

Supplement: Supplementary file 1 [file biomedicines-12-00308-s001.zip › Supplementary_Figure_S14.pdf]

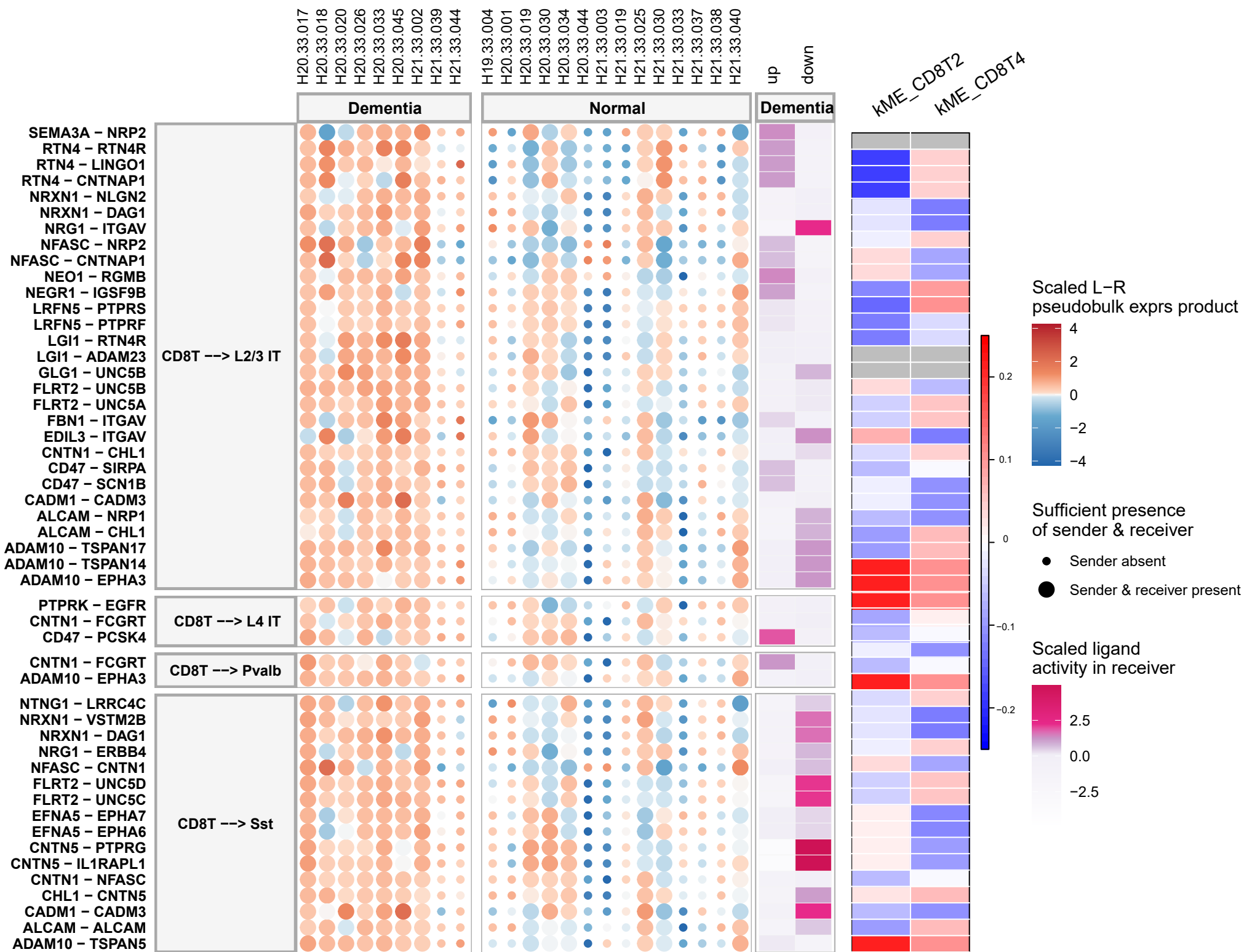

Supplement: Supplementary file 1 [file biomedicines-12-00308-s001.zip › Supplementary_Figure_S15_1.pdf]

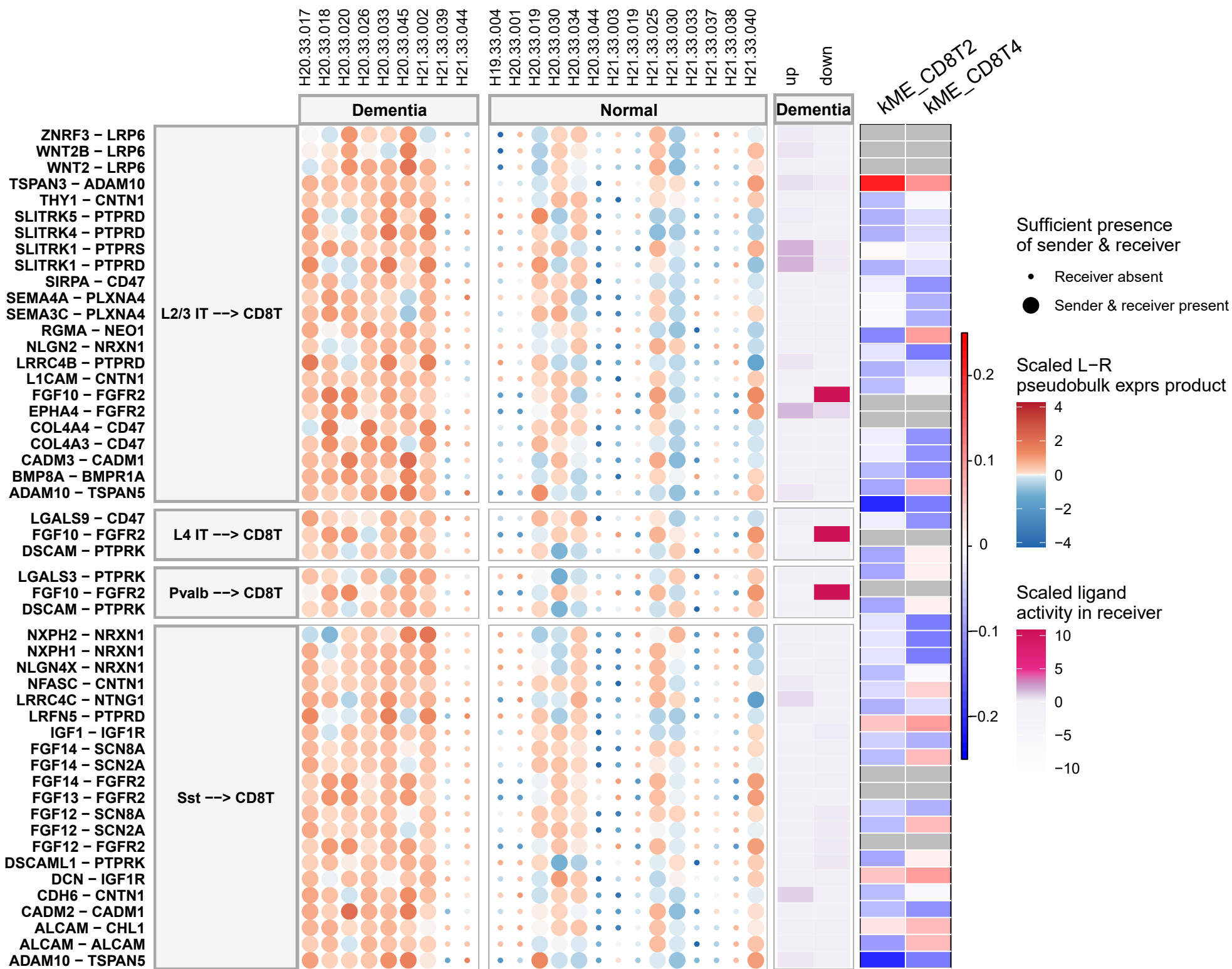

Supplement: Supplementary file 1 [file biomedicines-12-00308-s001.zip › Supplementary_Figure_S15_2.pdf]

Dementia

Cognitively Normal

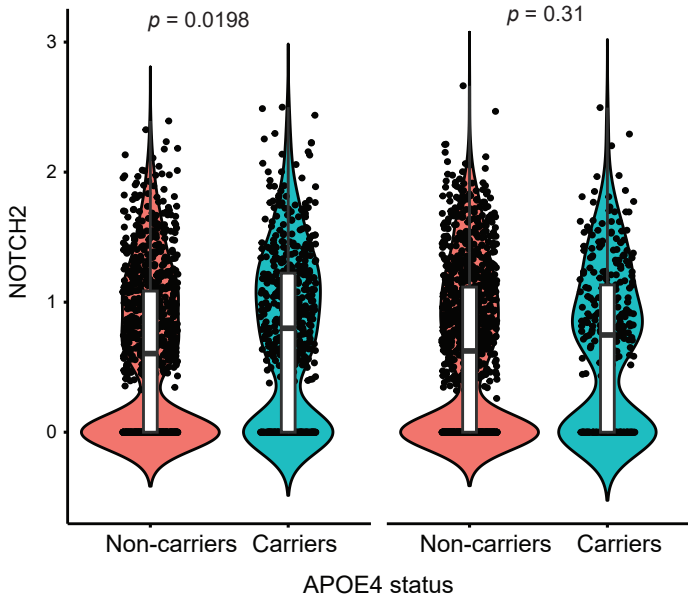

Supplement: Supplementary file 1 [file biomedicines-12-00308-s001.zip › Supplementary_Figure_S16.pdf]

Seurat clusters

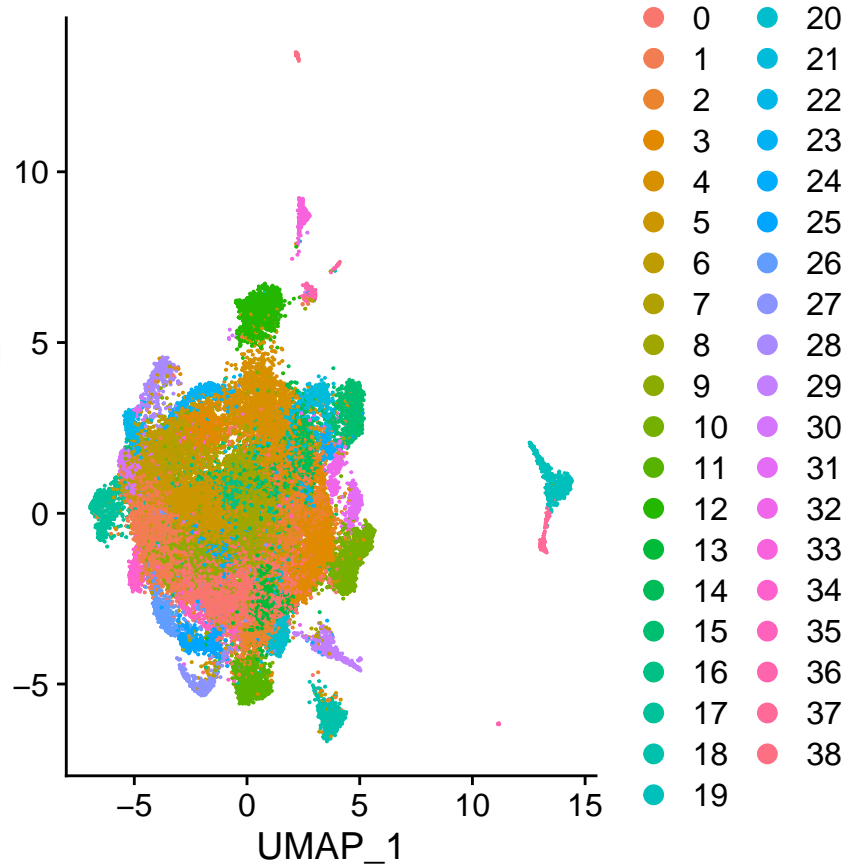

Age at death

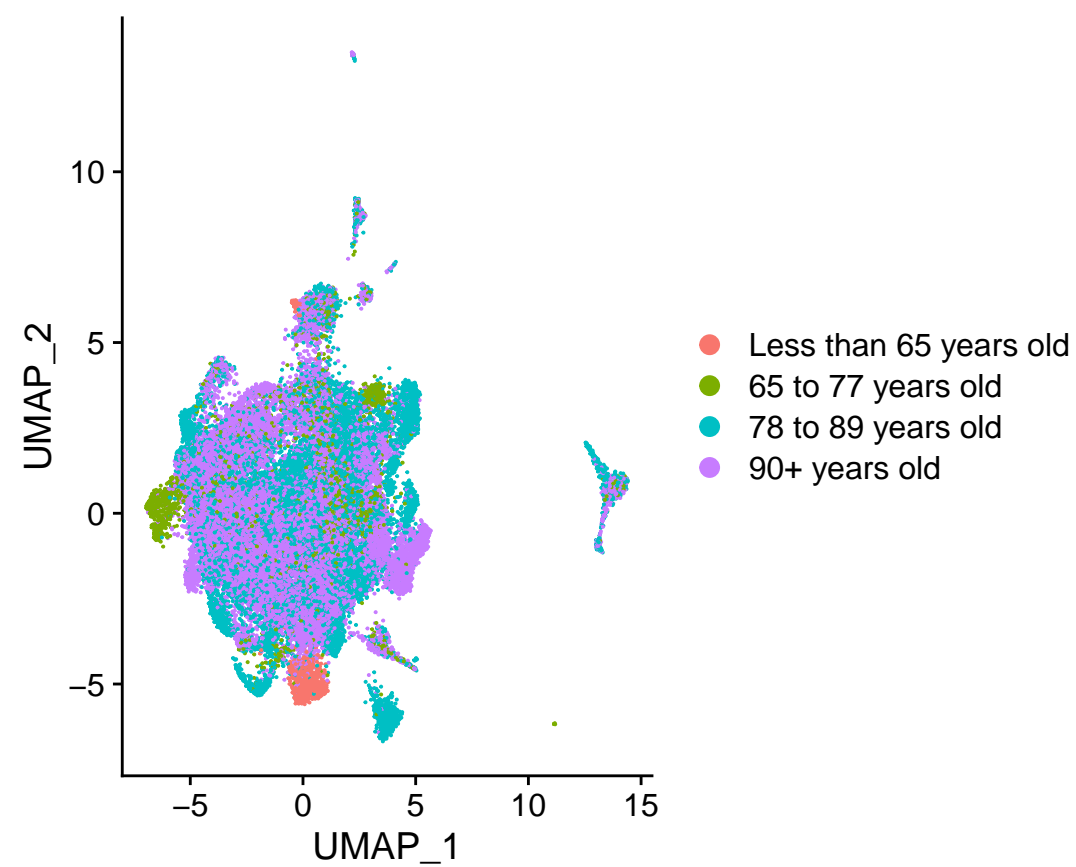

Donor ID

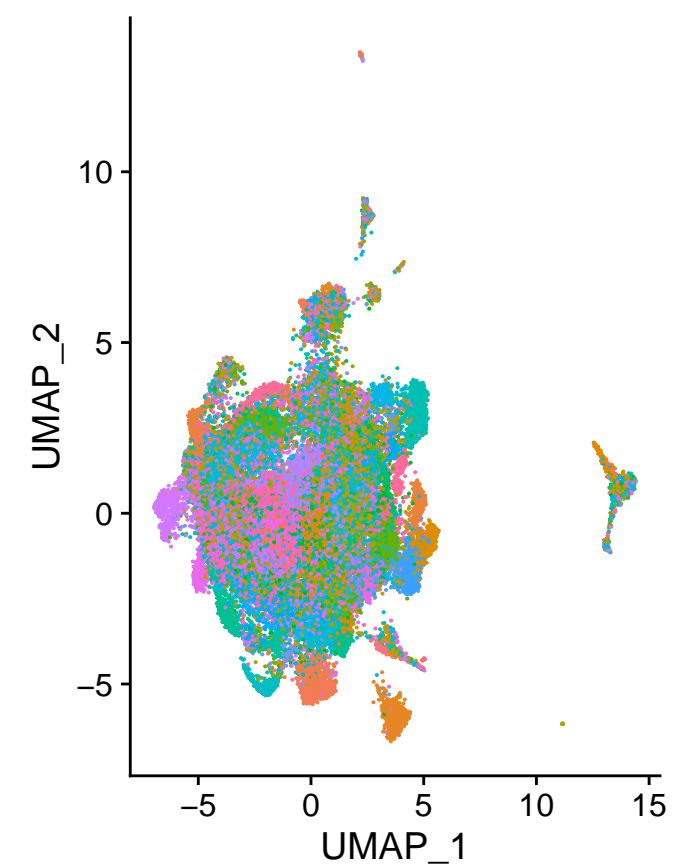

Specimen ID

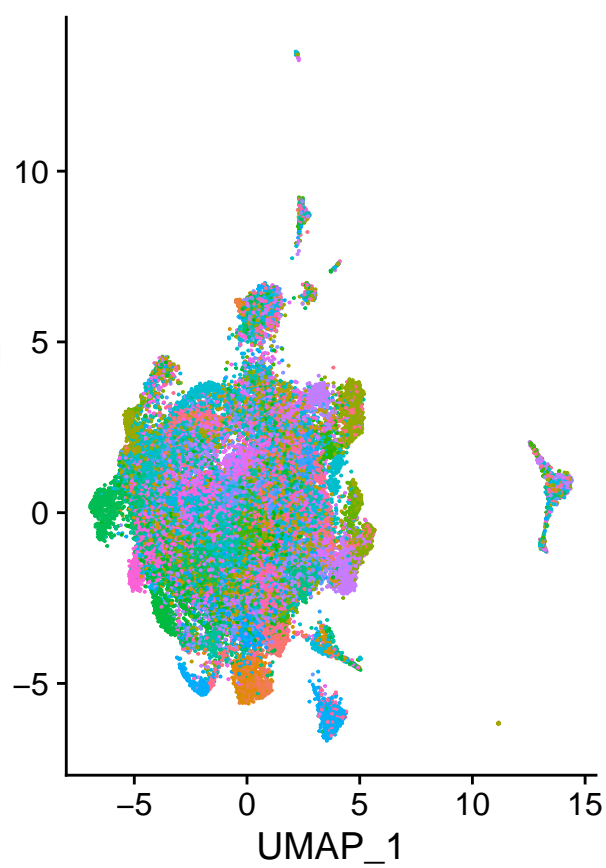

PMI

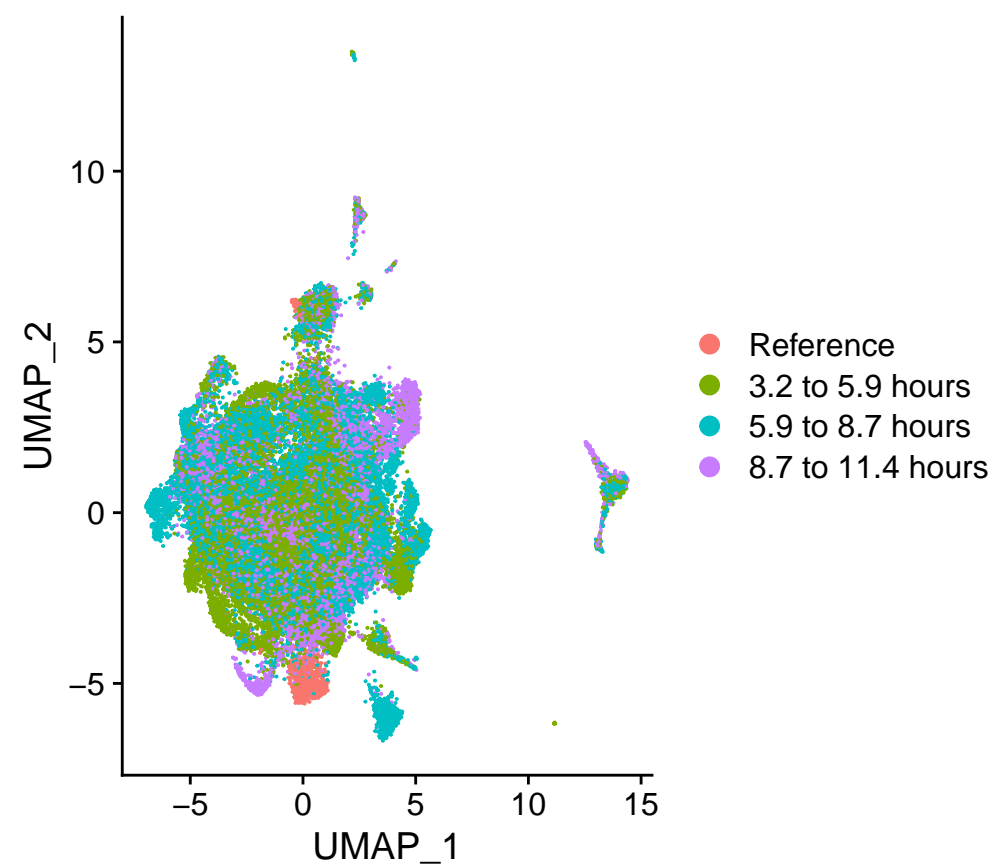

Sex

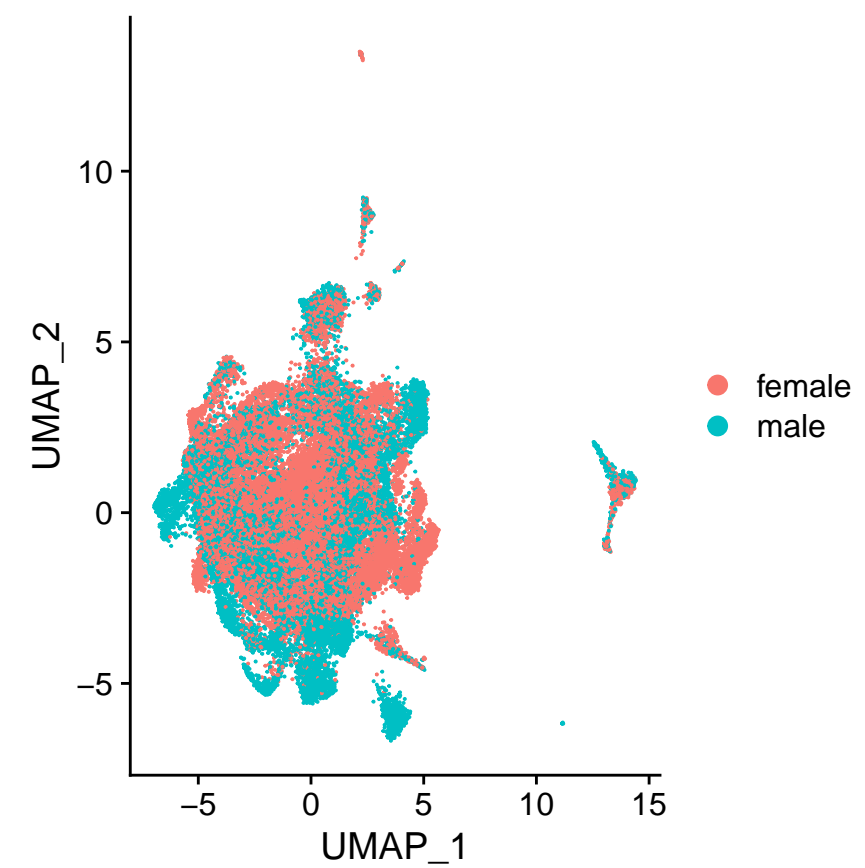

Supplement: Supplementary file 1 [file biomedicines-12-00308-s001.zip › Supplementary_Figure_S2.pdf]

**A**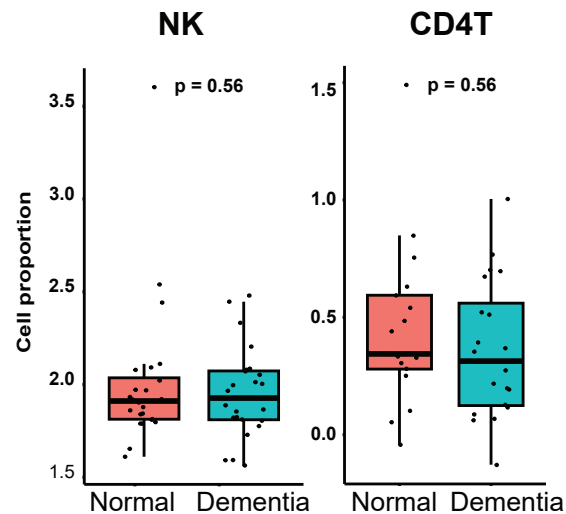**B**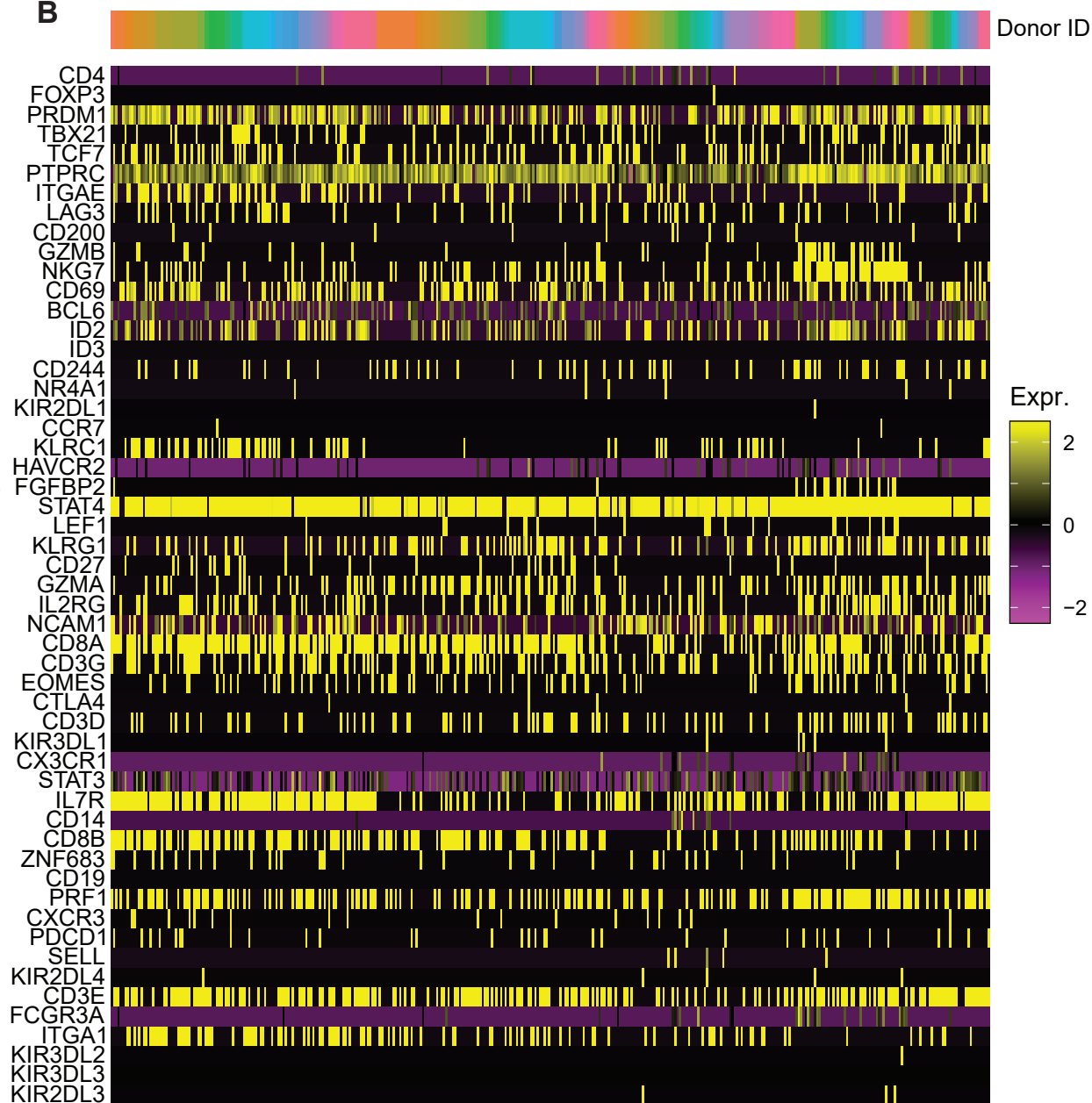

Supplement: Supplementary file 1 [file biomedicines-12-00308-s001.zip › Supplementary_Figure_S3_AB.pdf]

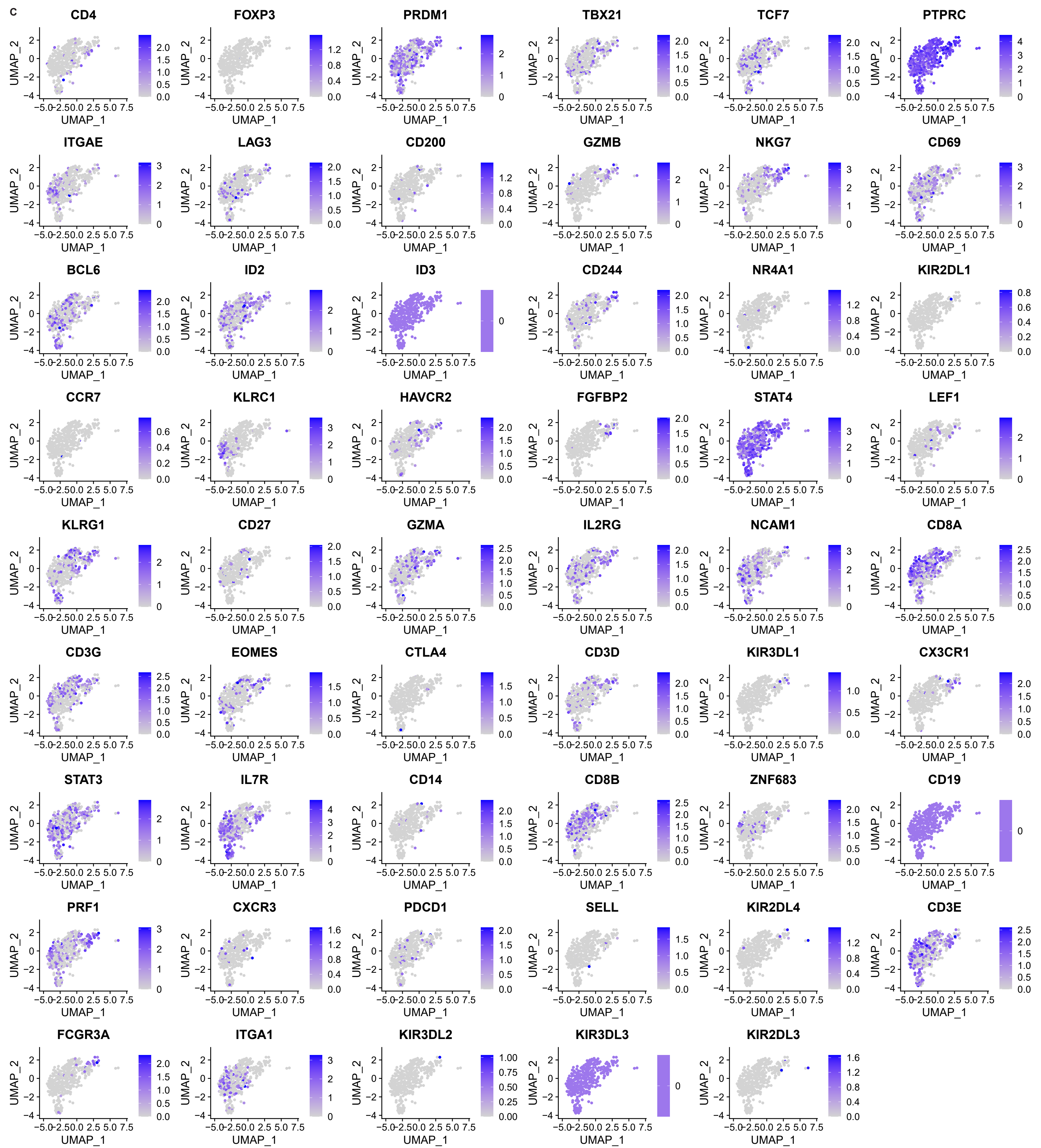

Supplement: Supplementary file 1 [file biomedicines-12-00308-s001.zip › Supplementary_Figure_S3_C.pdf]

A

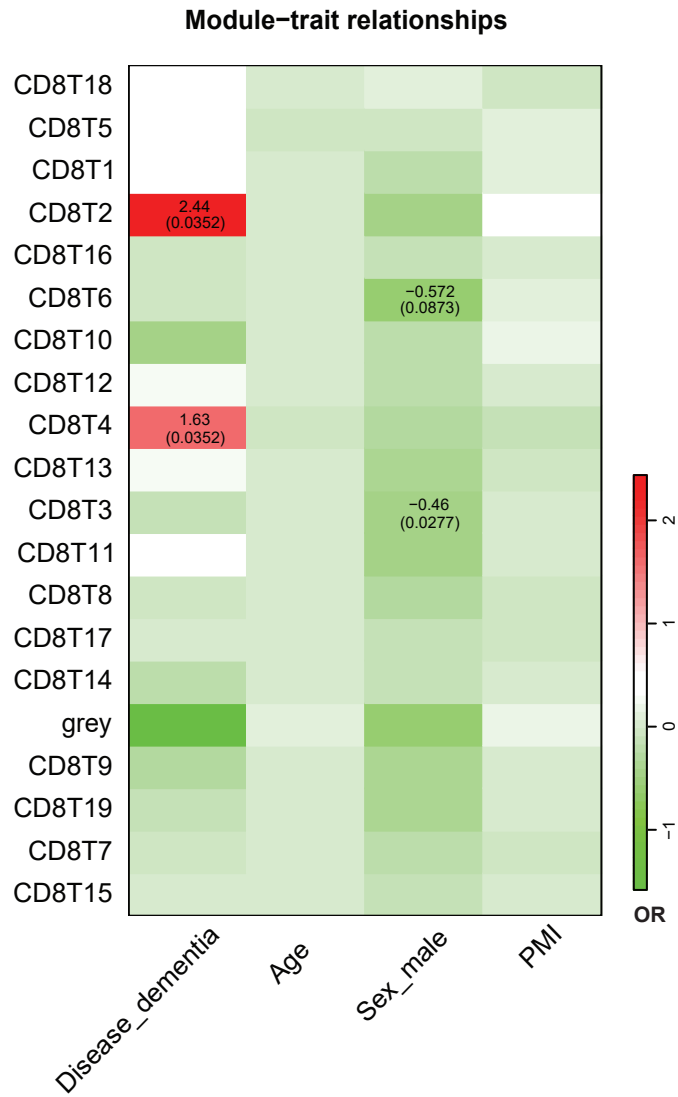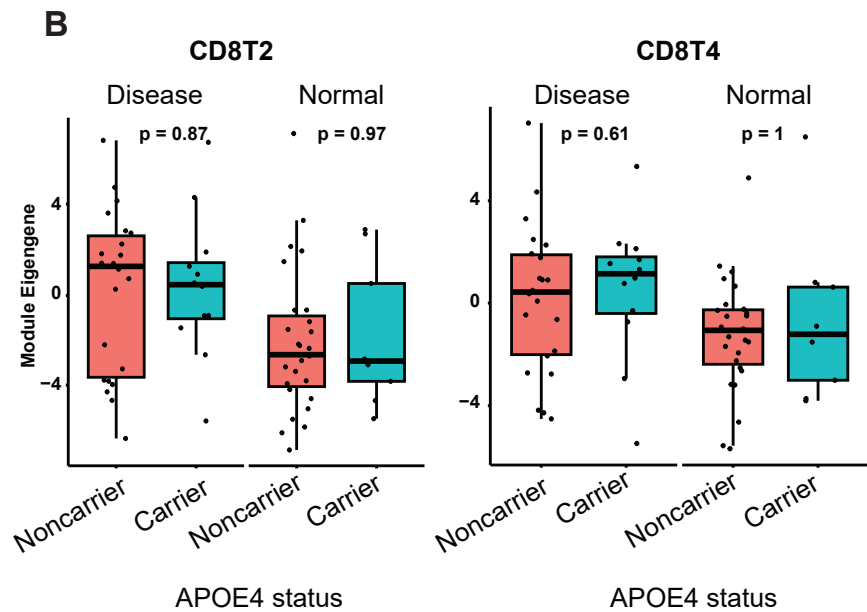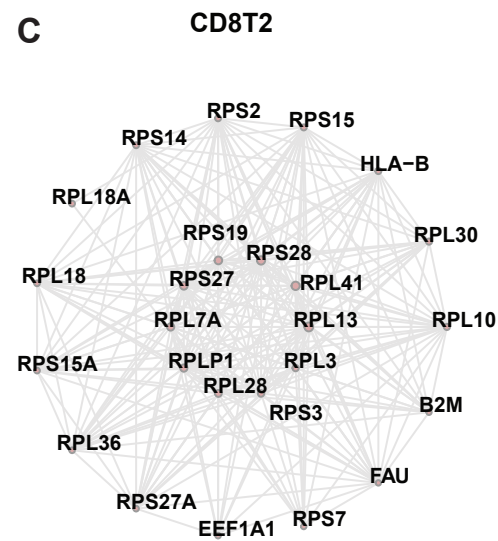

Supplement: Supplementary file 1 [file biomedicines-12-00308-s001.zip › Supplementary_Figure_S4.pdf]

**A** Module-trait relationships

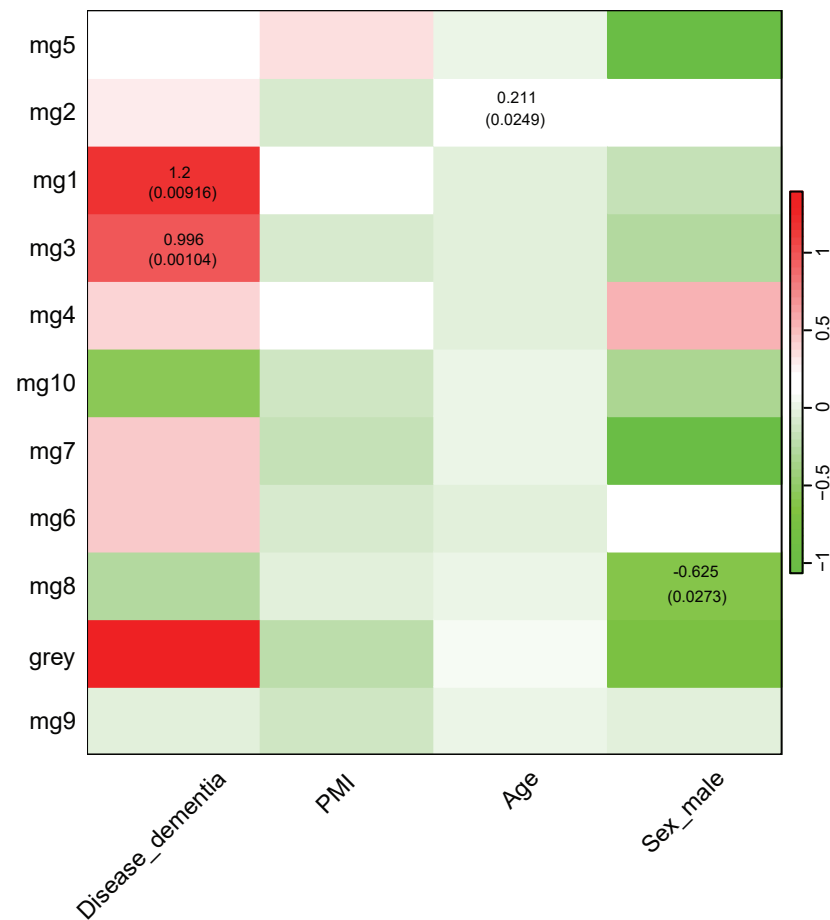

**B**

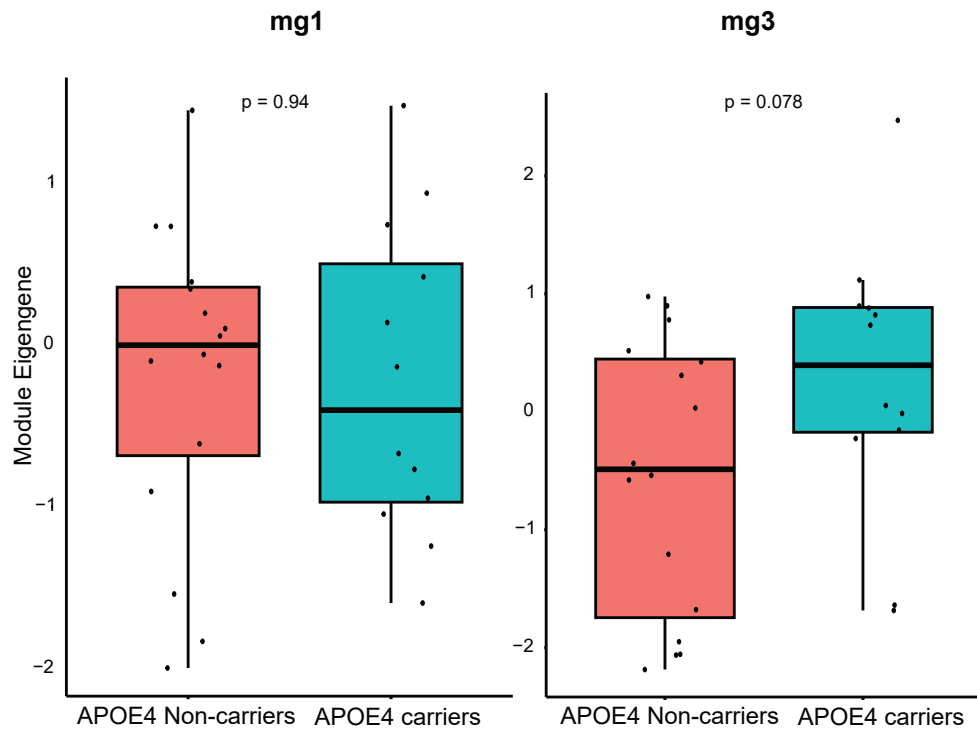

Supplement: Supplementary file 1 [file biomedicines-12-00308-s001.zip › Supplementary_Figure_S5.pdf]

**A**

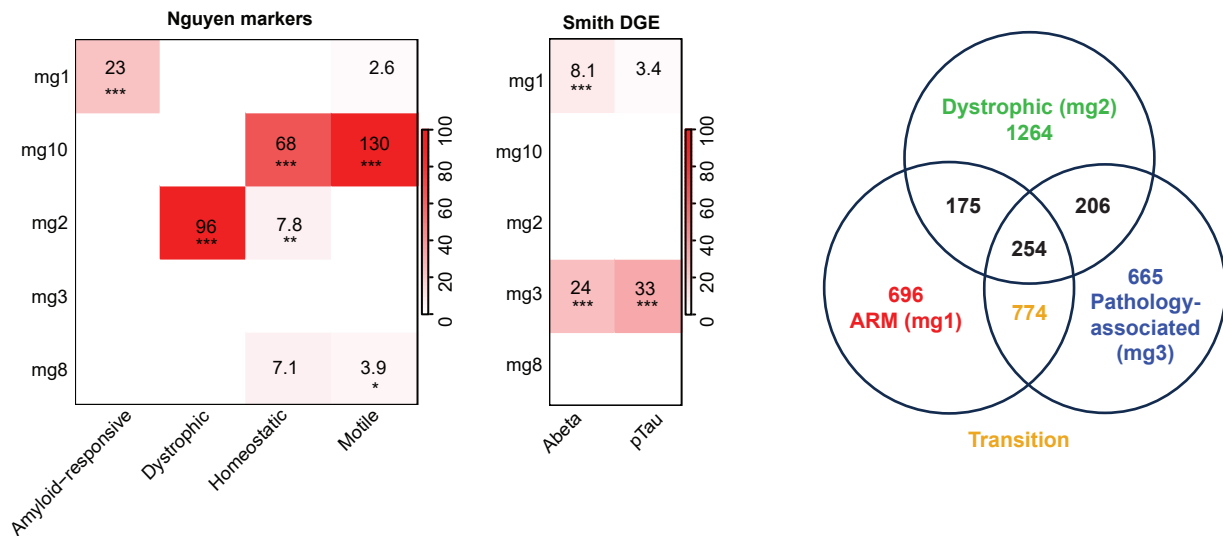

**B**

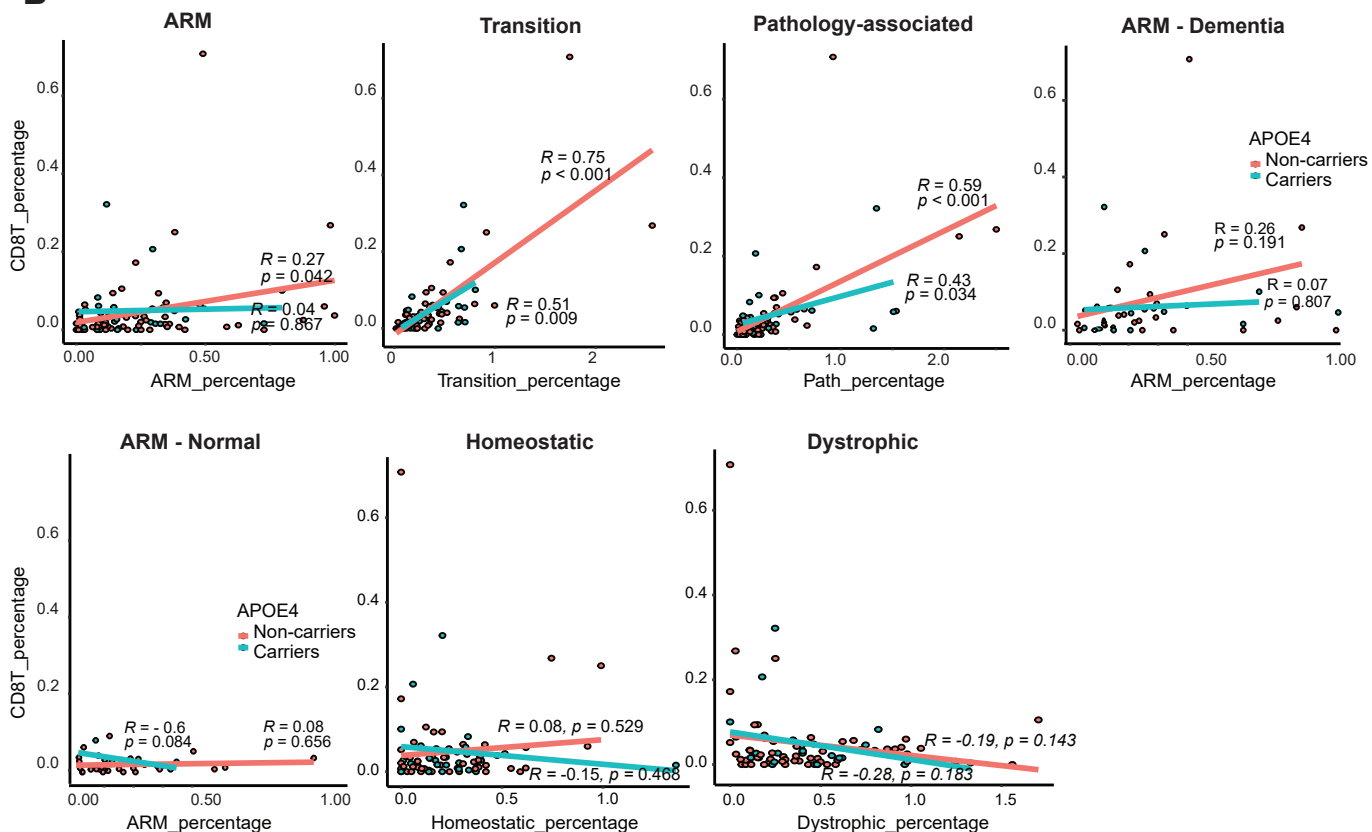

Supplement: Supplementary file 1 [file biomedicines-12-00308-s001.zip › Supplementary_Figure_S7.pdf]

# Cell type a bundances per sample

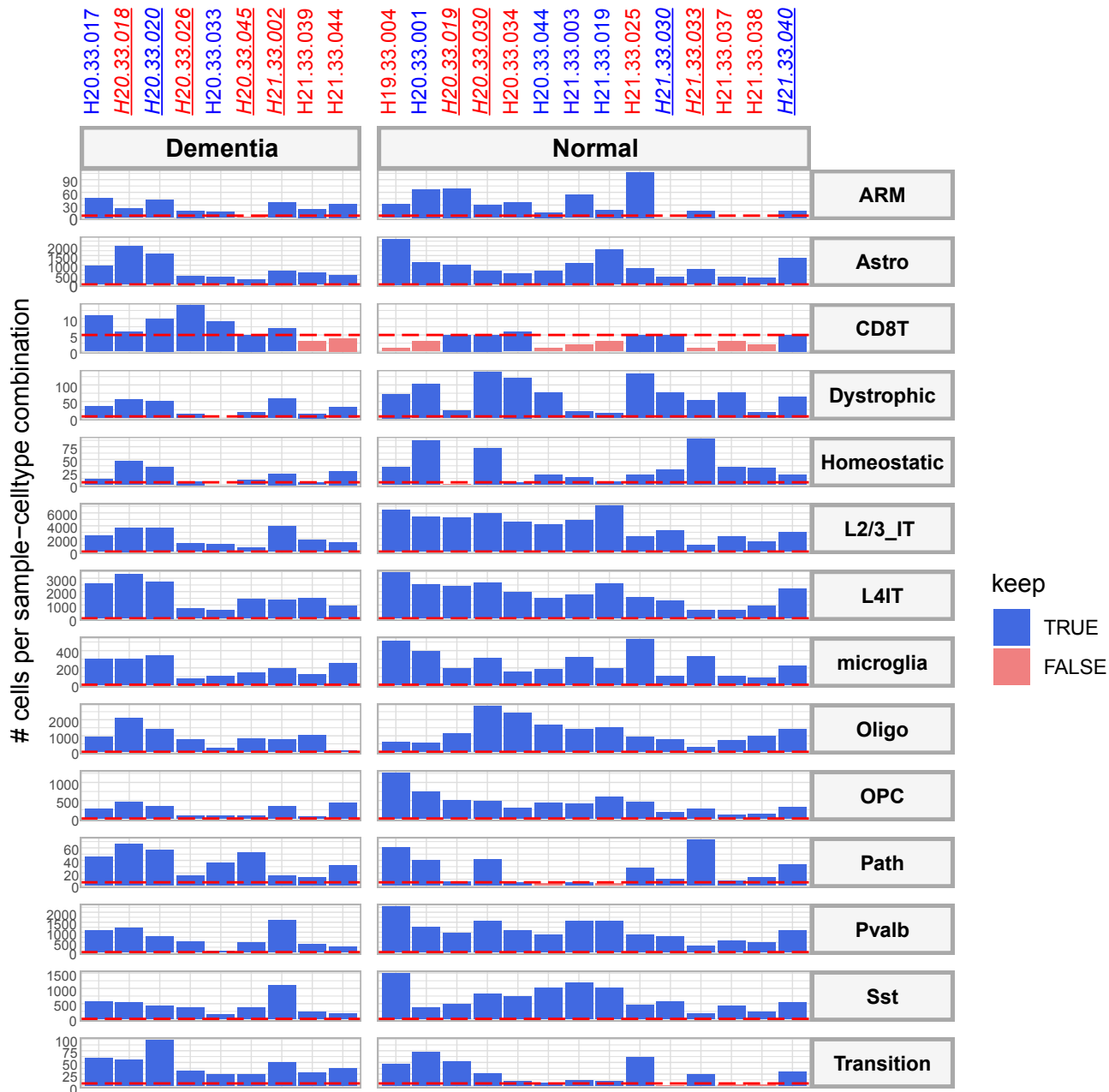

Supplement: Supplementary file 1 [file biomedicines-12-00308-s001.zip › Supplementary_Figure_S8.pdf]

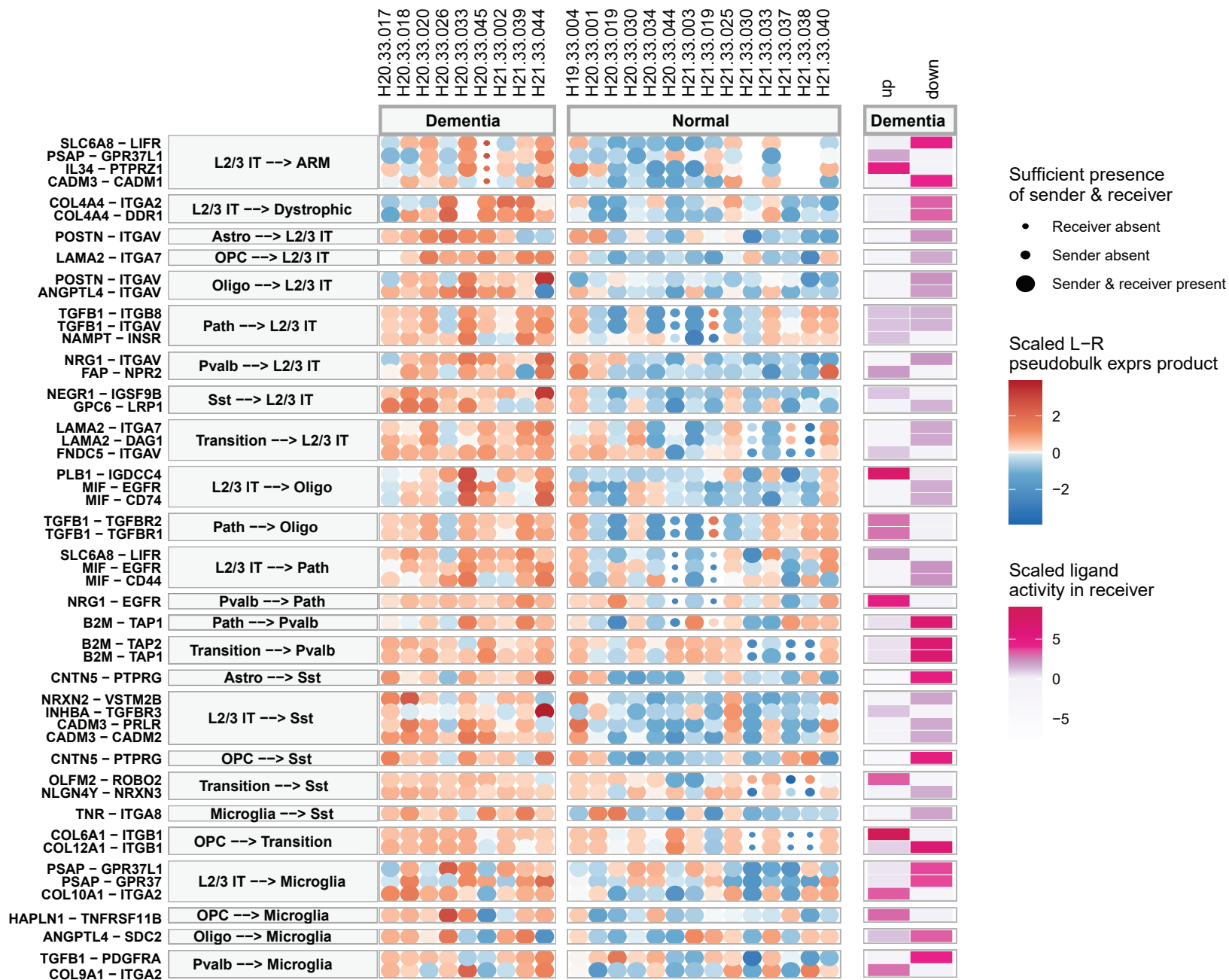

Supplement: Supplementary file 1 [file biomedicines-12-00308-s001.zip › Supplementary_Figure_S9.pdf]
